# The Emotion-to-Music Mapping Atlas (EMMA): A systematically organized online database of emotionally evocative music excerpts

**DOI:** 10.3758/s13428-024-02336-0

**Published:** 2024-01-30

**Authors:** Hannah Strauss, Julia Vigl, Peer-Ole Jacobsen, Martin Bayer, Francesca Talamini, Wolfgang Vigl, Eva Zangerle, Marcel Zentner

**Affiliations:** 1 Department of Psychology, University of Innsbruck, Universitätsstrasse 15, 6020 Innsbruck, Austria; 2 Department of Computer Science, Universität Innsbruck, Innsbruck, Austria

**Keywords:** Database, Emotion, GEMS, Music genres, Music Information Retrieval (MIR)

## Abstract

Selecting appropriate musical stimuli to induce specific emotions represents a recurring challenge in music and emotion research. Most existing stimuli have been categorized according to taxonomies derived from general emotion models (e.g., basic emotions, affective circumplex), have been rated for perceived emotions, and are rarely defined in terms of interrater agreement. To redress these limitations, we present research that served in the development of a new interactive online database, including an initial set of 364 music excerpts from three different genres (classical, pop, and hip/hop) that were rated for felt emotion using the Geneva Emotion Music Scale (GEMS), a music-specific emotion scale. The sample comprised 517 English- and German-speaking participants and each excerpt was rated by an average of 28.76 participants (SD = 7.99). Data analyses focused on research questions that are of particular relevance for musical database development, notably the number of raters required to obtain stable estimates of emotional effects of music and the adequacy of the GEMS as a tool for describing music-evoked emotions across three prominent music genres. Overall, our findings suggest that 10–20 raters are sufficient to obtain stable estimates of emotional effects of music excerpts in most cases, and that the GEMS shows promise as a valid and comprehensive annotation tool for music databases.

**Supplementary Information:**

The online version contains supplementary material available at 10.3758/s13428-024-02336-0.

## Introduction

The study of music-evoked emotion has grown in recent years into a rapidly expanding field at the crossroads of psychology, neuroscience, biology, musicology, advertising, music information retrieval, machine learning, and music recommender systems. Research within these areas typically uses excerpts from Western art music, pop, and film music, which last between 20 seconds and 2 minutes (Warrenburg, 2021). Although the number of music excerpts annotated for mood or emotion has grown considerably (Warrenburg, 2021), the benefits of this development are tempered by several factors. Specifically, information interrater agreement in emotional characterizations of music excerpts is often missing and content validity is questionable due to the reliance on emotion models that were not developed for describing aesthetic experiences. Furthermore, online music databases are often cumbersome to navigate. In the current study, we propose a number of research steps that can help redress these issues. On this basis, we initiate a new online database that differs from previous ones in several respects. In what follows, we discuss some of the most important aspects surrounding each of the above-mentioned issues.

### Interrater agreement in music-evoked emotion

In experimental psychology and neuroscience, the selection of music excerpts for emotion-inducing purposes usually does not follow a rigorous decision process and may be based on a variety of sources. These include expert opinion (e.g., Bigand et al., 2005; Eerola & Vuoskoski, 2011; Robazza et al., 1994), use in previous research (e.g., Lin et al., 2010; Mitterschiffthaler et al., 2007; Sammler et al., 2007; Schellenberg et al., 2008), pilot work (e.g., Altenmüller, 2002; Pêcher et al., 2009; Roy et al., 2008; Stephens et al., 2010; Witvliet & Vrana, 2007), the authors’ own research (e.g., Koelstra et al., 2011; Peretz, 1998), or a combination of the above (e.g., Kreutz et al., 2008; Ritossa & Rickard, 2004). In other cases, stimuli are composed by the authors themselves (e.g., Eerola et al., 2013; Gosselin et al., 2005; Koelsch et al., 2006; Lundqvist et al., 2009; Morton & Trehub, 2007; Vieillard et al., 2008). Such a variety of sources is not necessarily problematic as long as the decision for selecting emotionally evocative music excerpts can be pinned to a well-understood metric of effectiveness.

For example, the reliability with which a given piece of music may induce a certain emotion can be expressed by a widely understood effect-size metric, such as *r* or *d*, which describes the extent to which the music induces a particular emotion compared with that in a control condition. However, because music tends to elicit multiple or mixed emotional responses (e.g., Hunter et al., 2010; Larsen & Stasny, 2011; Zentner et al., 2008), it is sometimes not so much the induction of one particular emotion or mood that is of interest to researchers, but the pattern of evoked emotions. In that case, the question of effectiveness is more appropriately framed in terms of agreement in response patterning rather than in binary terms. For example, the effectiveness of an excerpt supposed to elicit a pattern of high arousal, high potency, and negative valence is best captured by an evaluation of the expected mean pattern in combination with a measure of interrater agreement, such as the intraclass coefficient (ICC) or Cohen’s kappa, rather than by *r* or *d*.

Information about the consistency in emotion ratings of music excerpts is not only important for purposes of emotion induction in experimental studies, but it also plays a critical role in work that uses signal processing and machine (deep) learning methods to characterize or predict musical emotions, such as music emotion recognition (MER; e.g., Gómez-Cañón et al., 2021; Gómez-Cañón et al., 2022; Han et al., 2022; Kim et al., 2010; Panda et al., 2023; Yang & Chen, 2011a). In work seeking to apply machine learning to music and emotion, emotion annotations of music by humans are essential for training machine learning algorithms for classification or recognition of music emotions.

It should be recognized, however, that several sources of error can compromise the validity of human annotations (Gómez-Cañón et al., 2021), directly impacting and limiting the performance, validity, and reliability of MER systems. Prime among these limitations is low interrater agreement (Lange & Frieler, 2018; Schedl et al., 2018), particularly when it comes to describing fine-grained emotions due to interindividual variability in the subjective perception of nuances in emotional experience (Juslin & Laukka, 2004).

### Describing music-evoked emotion

The validity of characterizations of music-evoked emotions is contingent on the particular set of mood or emotion descriptors that investigators select for describing music-evoked emotions. Initially, research on music and emotion relied on emotion terms derived from general emotion models that were developed to characterize everyday emotions rather than music-evoked emotions. For example, ratings of music-evoked emotion have often relied on the *circumplex model* of emotion, which posits that all emotions can be represented as points in a two-dimensional space defined by arousal, valence, and, more rarely, potency (Zentner & Eerola, 2010).

Because of its parsimony, the dimensional approach is frequently used in MER research and has also been used in a number of datasets of music excerpts with emotion annotations (see Zhang et al., 2018), such as the 1000 Song Database (Soleymani et al., 2013), 4Q audio emotion dataset (Panda et al., 2020), AMG1608 (Chen et al., 2015), the Database for Emotional Analysis in Music (DEAM, see Aljanaki et al., 2017), the Database for Emotion Analysis using Physiological Signals (DEAP, see Koelstra et al., 2011), the Greek Music Dataset (GMD, see Makris et al., 2015), MER60 (Yang & Chen, 2011b), MoodSwings (Speck et al., 2011), the Popular Database with Emotional Annotations (PMEmo, see Zhang et al., 2018), and SoundTracks (Eerola & Vuoskoski, 2011). 

The simplicity and elegance of the circumplex model are offset by two important limitations. First, the circumplex approach is a general model of affect and was not devised to capture the distinctive features of music-evoked emotions. Second, the model lacks granularity. For example, fear and anger, two evidently distinct emotions, occupy a similar position in the affect space defined by the circumplex model. Moreover, there are several positive-valence and low-arousal emotions among those elicited by music (e.g., love-tenderness, nostalgia), whose important qualitative differences are lost in the affective circumplex.

Characterizations of music-evoked emotion have also relied on concepts from basic emotions theory (Ekman, 1992a), notably happiness, sadness, fear, and anger, sometimes eclectically amplified by concepts that are not part of basic emotions but seem musically plausible to the researchers, such as tenderness or awe (Zentner & Eerola, 2010; Juslin et al., 2016). Examples for datasets with annotations inspired by basic emotions theory include Emotify (Aljanaki et al., 2016), MagnaTagATune (Law et al., 2009), Primary Musical Cues (Eerola, 2016; Eerola et al., 2013), SoundTracks (Eerola & Vuoskoski, 2011), and TROMPA [Towards Richer Online Music Public-domain Archives]-MER (Gómez-Cañón et al., 2022). The main limitation of basic emotions theory is that it was conceived to account for survival-type emotions such as anger, fear, or disgust, which Scherer and Zentner (2008) characterized as “utilitarian” as opposed to “aesthetic emotions”. Although it has been found that music is able to communicate some of the basic emotions, their relevance for describing felt emotions is limited. Thus, while music may sound angry, it is only very rarely that music is capable of inducing genuine anger (Zentner et al., 2008; Juslin et al., 2016).

For researchers, relying on well-known and comparatively parsimonious models has obvious benefits. For one, the limited choice of emotion or affect terms offered by basic emotions or circumplex approaches allows annotators to rate music quickly, thereby making data collection more efficient (Juslin & Laukka, 2004). Data analysis and computations may also be easier, especially in fields that apply machine learning approaches to music emotion recognition. For instance, in a very simple categorical model that uses the two categories “happy” and “sad”, the chances of randomly guessing the correct emotion in a balanced dataset is already 50%; whereas for more complex models of emotion, the task is more complex and computationally intensive. However, such simplification also means that much emotional information is missed and cannot be leveraged by machine learning approaches for optimizing prediction and classification.

In a series of studies examining the differential validity of various approaches to measuring musically evoked emotion, Zentner and colleagues (Zentner et al., 2008) found that the richness of musically inducible emotions goes beyond emotion terms and concepts provided by basic emotions theory, the affective circumplex, or other generic models of emotion, requiring a domain-specific approach instead. Specifically, the authors started out with 515 emotion terms, successively eliminating those terms that were rarely used to describe music-evoked emotions and retaining a few dozen core emotion terms, titled GEMS for Geneva Emotional Music Scale. Factor analyses of GEMS ratings for a variety of music revealed a hierarchical structure of music-evoked emotion (Zentner et al., 2008). This structure, which is sometimes referred to as the GEMS model, comprises three second-order and nine first-order factors: (1) sublimity (wonder, transcendence, tenderness, nostalgia, and peacefulness), (2) vitality (joyful activation and power), (3) unease (tension and sadness). In recent years, researchers have largely confirmed the factorial structure of the GEMS model (Chełkowska-Zacharewicz & Janowski, 2021) and recognized the importance of several GEMS dimensions for characterizing musically evoked emotions, such as wonder-awe, tenderness, or nostalgia (e.g., Barrett et al., 2010; Juslin, 2013; Trost et al., 2012).

Although the GEMS was derived from verbal reports of emotion, other sources of evidence have corroborated its validity. In one study, verbal reports of emotions obtained with the GEMS were found to correlate with emotionally relevant brain activation patterns (Trost et al., 2012). Another study found that music chosen to induce specific GEMS emotions led to better recall of pictures when they were congruent with rather than incongruent with the emotions conveyed by the music (Talamini et al., 2022). Both studies converge in showing that the emotions captured by the GEMS are more than mere expression of verbal-semantic representations.

Although these findings indicate that the GEMS holds promise as a system for characterizing and classifying emotional effects in music information retrieval (MIR), there were also some limitations in the original work that call for additional research. Specifically, the music excerpts used in the original study were not systematically balanced with regard to genres and subgenres, and this could have led to a biased representation of core musical emotions. Furthermore, the original publication of the GEMS was based on French-speaking samples, and the use of the GEMS in other population and language groups remains under-researched. Some investigators have suggested adding terms that they felt were missing in the original version of the scale (e.g., Aljanaki et al., 2016; Coutinho & Scherer, 2012), but it is unclear whether such additions are empirically warranted. Thus, evaluating the merits of the GEMS on the basis of a more diverse sample of music excerpts and of music listeners is an important step to take.

To our knowledge, two projects have collected emotion ratings of music excerpts using the GEMS. In their Emotify database, Aljanaki and colleagues (Aljanaki et al., 2016) provide a publicly available collection of GEMS-rated music excerpts. The authors used a crowdsourcing game to collect ratings for 400 songs, randomly selected from the genres pop, rock, classical, and electronica. Each song was annotated by an average of 20 raters. Both sound files and annotations can be downloaded via the project webpage^1^. One limitation of this project is that participants were provided with only nine labels, reflecting the nine GEMS categories, rather than the full spectrum of GEMS labels, and instructed to select a maximum of three GEMS categories per song, to be rated as either present or absent (Aljanaki et al., 2016). Although these changes are understandable, they also meant that the benefits that the GEMS offers in nuance over that of other approaches could not be fully exploited.

In the PMEemo database (Zhang et al., 2018), ratings of the nine GEMS dimensions and on valence and arousal were obtained for a total of 749 song choruses drawn from music charts from 2016 to 2017, with durations ranging from 11 to 88 seconds, provided by 457 subjects. Although the information is available in the form of a publicly accessible spreadsheet on the project webpage^2^, it does not include values for the GEMS dimensions, but only for valence and arousal.

### Perceived versus felt emotion

Another potential source of validity issues relates to the distinction between perceived emotion and induced emotion. Perceived emotions are those that the music is believed to express or portray; they essentially represent an emotional characterization of the music. Hence, the respective ratings are likely to primarily engage cognitive and associative processes. In contrast, induced emotions refer to what listeners themselves feel in response to the music. Consequently, the respective ratings primarily relate to experienced changes in feeling tone. Although the two modes of responsiveness tend to converge in everyday emotion-eliciting contexts (e.g., a situation perceived to be frightening usually does generate fear), in aesthetic contexts, the two modes of emotional responsiveness often diverge. Thus, music that is generally perceived to express fear or sadness can nevertheless be enjoyed on the whole (e.g., Eerola et al., 2018).

One difficulty with previously rated music excerpts is that in 75% of relevant studies, music excerpts were rated for perceived rather than felt emotions (Warrenburg, 2021). Hence, the sample of excerpts rated for induced or felt emotion is relatively small (Eerola & Vuoskoski, 2013; Gómez-Cañón et al., 2021), and what is available has often been annotated using basic emotions or the affective circumplex, whose limitations we described earlier. Another difficulty is that the distinction between the two modes of emotional responsiveness is not always clearly drawn. For example, a semantic affect space that bears some resemblance to the GEMS was proposed by Cowen et al. (2020). Within the bounds of this space, the researchers provide a fascinating interactive map of 2000 5-second excerpts, which were rated by 2777 US and Chinese participants, and are made freely available to users. As is the case with the GEMS, the classification transcends basic emotion models and includes dimensions such as “joyful/cheerful”, “energizing/pumped-up”, “calm/serene”, “dreamy”, “erotic/desirous”, “sad/depressed”, and “tense/anxious”. However, the assessments did not explicitly differentiate perceived from felt emotion. This may help explain the greater number of negative affect categories relative to the GEMS, or the presence of the category “beautiful”, which seems better suited to describe properties of the music than features of an experienced emotion.

### Structure and organization of databases

A fourth and final challenge for researchers is that in most current music collections and databases, information on emotional effects of music excerpts is often stored in unwieldy formats. Sometimes the information is being spread across multiple csv files that researchers need to piece together to obtain the desired information, for example, in the PMEmo database (Zhang et al., 2018) or the DEAM database (Aljanaki et al., 2017). Screening for emotionally suitable music excerpts could be greatly facilitated if the information were concentrated in one place, including tabs that allow users to select music excerpts according to important criteria, such as type of emotion, profile of emotions, features of the music (such as vocal vs instrumental), genre, and period, as well as to request information on the source, type, or reliability of the emotion ratings. Sometimes, such as when several excerpts are needed to induce a given emotion in repeated-measures designs, or when suitable control stimuli are needed, users may also wish to be able to search for excerpts with similar, dissimilar, or unrelated emotion profiles. However, retrieval of music excerpts along such criteria is typically onerous.

### The present research

To redress some of the aforementioned issues and gaps, we aimed at creating a database that would include (a) a large variety of vocal and instrumental excerpts from mainstream music genres, (b) excerpts that are rated unambiguously for induced rather than perceived emotion, (c) emotion ratings that are based on a validated taxonomy of music-evoked emotion, and d) a clear organization that facilitates search for excerpts according to criteria such as musical features, emotional effects, familiarity, and interrater reliability.

In view of achieving these objectives, we had a few hundred music excerpts from three distinct mainstream genres (classical, pop, hip-hop/rap) rated by between 10 and 40 listeners with the 45-item version of the GEMS. We included classical music because most studies on music and emotion focus on this genre (Eerola & Vuoskoski, 2013; Västfjäll, 2001; Warrenburg, 2020). Hip-hop/rap and pop music were chosen because they are the most popular genres on streaming platforms and radio airplay (see Viberate, 2021; IFPI, 2023), and the three genres were different enough to provide a good test of the GEMS’ range of application.

In preparing the database, we ran a number of analyses to address the validity of our approach. First, it is unknown at present how many raters are needed to obtain stable estimates of the emotions evoked by music excerpts. In analyses addressing this question, we examined the relationship between number of raters and the stability of the resulting emotion annotations. Second, to serve as an efficient and valid tool for annotating music-evoked emotions, the GEMS ought to be neither over-inclusive nor under-inclusive. The GEMS would be over-inclusive if it included too many labels—labels for emotions that are only rarely used by listeners to describe their emotional responses to music. Over-inclusiveness would lessen the efficiency of the GEMS. To examine potential over-inclusiveness, we analysed frequencies and intensities of individual GEMS items and dimensions and examined their salience across the three music genres. Conversely, the GEMS would be under-inclusive if it left out terms that are critical for characterizing music-evoked emotions. Under-inclusiveness would lessen the validity and, hence, the range of application of the GEMS. To assess potential under-inclusiveness, we gave listeners the opportunity to add emotion terms they felt were missing from the GEMS. We also assessed a number of personal characteristics of the raters and examined their effect on the ratings they provided. Finally, the most important features of the resulting new database are described.

## Method

### Sample

Participants were recruited via a university mailing list, the crowd-sourcing platform Prolific, and postings on the social media platforms Facebook, Instagram, and Reddit. Psychology students from the University of Innsbruck (*n* = 92, 16.2%) received course credit for their participation, whereas participants recruited via Prolific (*n* = 290, 51.1%) received 6 GBP. Participation via Prolific was restricted to subjects reporting English as their first language.

A total of 567 participants provided complete ratings. Of those, five individuals (0.9%) were partially or fully missing information on language version and socio-demographic data due to technical issues. About half of the participants completed the assessment in English (*n = *306, 54.0%) and half in German (*n* = 258, 45.5%). Overall, 96.8% of the participants reported being fluent in the language they participated in. A total of 316 participants (55.7%) identified as female, 243 (42.9%) as male, and 5 (0.9%) as other. Participants’ age ranged from 16 to 76 years (*M = *28.70, *SD* = 10.35, *Mdn* = 25). The vast majority of the sample identified as being from Western nations, with the most common nations being Germany (31.7%), the United Kingdom or the Republic of Ireland (21.3%), and the United States (20.5%). The remaining participants either represented various other nationalities (21.2%) or provided no valid information (5.4%). Most of the participants (80.8%, *n* = 458) reported either being currently enrolled in or having completed some kind of university studies. More detailed descriptive statistics for both English- and German-speaking participants can be found in Table S1.

### Stimuli

Music stimuli were selected by a dedicated panel consisting of (a) three staff members with extensive knowledge of music and emotion research and 10 or more years of music training and (b) six undergraduate students with strong involvement in music as musicians, producers, listeners, or all three combined. An important consideration in selecting the excerpts was that they should represent a relatively broad spectrum of music from each genre. In the case of classical music, for example, we strove to include music (a) from each of the major canonical periods (baroque, classic, romantic, modern), (b) that was instrumental and/or vocal, and (c) that varied along the dimensions of arousal and valence. Similarly, the selection of pop and rap/hip-hop songs aimed to represent music (a) from different decades (i.e., from the 1960s to current charts) and subgenres, (b) that was performed by both individuals and groups (including both males and females), and (c) that also varied along the dimensions of arousal and valence.

Whereas all pop excerpts comprised vocals, hip-hop/rap and classical music excerpts were derived from pieces with and without vocals. In particular, instrumental-only pieces from the instrumental hip-hop/lo-fi subgenre accounted for about a fifth of the excerpts for hip-hop/rap, whereas for classical music about a fourth of the pieces included vocals (i.e., soprano, tenor, or choir). In addition, as we targeted both English- and German-speaking raters, music excerpts were chosen to represent both languages, with German lyrics making up 15.8% of all hip-hop/rap excerpts and 3.6% of all pop excerpts. Applying these criteria, we identified 364 music pieces in total: (1) classical music (*n* = 105), (2) hip-hop/rap (*n* = 120), and (3) pop (*n* = 139).

From the chosen music, our panel extracted excerpts lasting between 30 and 60 seconds that were deemed particularly characteristic of the respective music pieces. The essence of the pop and hip-hop/rap songs could generally be captured in less time than that of classical music pieces due to frequent repetitions in the songs; hence, average durations of the musical excerpts were slightly longer for the classical pieces (*M* = 47 seconds, *SD* = 10, *Mdn* = 46) than for pop (*M* = 37 seconds, *SD* = 8, *Mdn* = 34) and hip-hop/rap (*M* = 40 seconds, *SD* = 7, *Mdn* = 39). A similar pattern of differences in duration between Western art music and other genres can be found in most of the previously used music stimuli (for a review, see Schubert, 2013; Warrenburg, 2020).

### Measures

#### Socio-demographic data

Participants were asked several questions regarding their socio-demographic background, including gender, age, nationality and language proficiency, education, and work activity.

#### Geneva Emotional Music Scale

Music excerpts were rated with the GEMS (Zentner et al., 2008). The GEMS is the consolidated form of emotion terms used in the final two studies of the original work (Zentner et al., 2008; see also Appendix). It contains 45 labels that can be grouped into nine different dimensions (*wonder, transcendence, tenderness, nostalgia, peacefulness, energy*^3^*, joyful activation, sadness,* and *tension*) and three higher-order factors (*sublimity, vitality,* and *unease*). In the current study, emotion terms were rated on a scale of 0 to 100. Non-selected items were set to 0 in line with the approach used in Study 3 by Zentner et al., 2008 (see also “Procedure” subsection). From these ratings, the mean intensities for the nine GEMS dimensions were computed for each music excerpt using Eq. 1 (Gerstgrasser et al., 2022).^4^ Unlike the case of an arithmetic mean value, the values resulting from the formula account for both the number of chosen emotion terms and their intensity.

Formula for the calculation of emotional intensity, where x̄ = mean intensity of the emotion dimension or in total, Σs = number of selected emotions, and N = number of emotions available for the emotion dimension or in total.

1 $$(\overline{x } +\frac{\overline{x} \times \sum s}{N })/2$$

Scores for the higher-order GEMS factors were derived by averaging across the respective dimensions. Internal consistency (*ω*) of the GEMS, computed across all excerpts and raters, ranged between .54 and .72 for the nine dimensions and between .64 and .79 for the three factors.^5^ From a review of the literature, the terms *bored* and *disgusted* were included to allow listeners to express lack of interest and dislike (Aljanaki et al., 2016; Coutinho & Scherer, 2012), but these terms were not included in the computation of dimensional or factor scores.

#### Other measures

Participants were also asked to indicate familiarity with the song and liking of the song on a scale ranging from 1 (*not at all*) to 5 (*very*). To examine the impact of annotators’ personal characteristics on their ratings of music-evoked emotions, we assessed music background, genre preferences (Rentfrow & Gosling, 2003), music listening motives (Chamorro-Premuzic & Furnham, 2007), personality traits (Körner et al., 2008; Paulus, 2009), and current mood state (Thompson, 2007). This part of the study is reported in an online Supplemental Appendix (henceforth SA).

### Data-analytic strategy

**Fig. 1:**
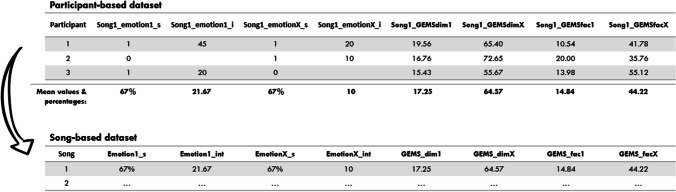
Visualization of the structure of the two datasets used for the analyses

Responses were organized into two different datasets, as illustrated in Fig. 1. The upper part of the figure shows data arranged by participants. Each row represents a participant, followed by the participant’s ratings of the music excerpts: first, the binary information about whether the person selected a given emotion from the GEMS (0 = *no*, 1 = *yes*, e.g., *Song1_emotion1_s*), and second, the felt intensity (e.g., *Song1_emotion1_i*). If a participant did not select an emotion term, we set the missing value to 0 in accordance with the instructions provided to participants (see Measures). 

The lower part of Fig. 1 shows arrangement of the data by songs. The arrow extending from the upper to the lower part of the figure indicates that GEMS values for each song were averaged emotion ratings computed across participants. The different column labels refer to the type of score and whether it relates to a GEMS dimension (e.g., *Song1_GEMSdim1*) or a GEMS second-order factor (*Song1_GEMSfac1*). Next to the columns for the emotion scores were columns that included mean ratings on familiarity and liking of the songs, as well as number of raters per song (not shown in Fig. 1). Finally, we coded order of presentation of the music excerpts so that the position of a piece could be correlated with the respective emotion ratings.

We used the participant dataset to address research questions about interindividual variability in music-induced emotions, for example, to examine interrater agreement and associations between annotator characteristics and music-induced emotions. The song dataset was used to determine the emotion profiles evoked by different songs and to display them in the online database together with other information on the music excerpts.

### Procedure

Music excerpts were allocated to sets of 10 to 12 excerpts for a total of 36 sets that were randomly administered to participants. Each set was balanced regarding genre, tempo, and overall impressions of emotional tone so that any participant would listen to a broad variety of music. Stimuli were delivered online with LimeSurvey (version 2.64.1+; LimeSurvey GmbH n.d.) and hosted on a server of the University of Innsbruck. Upon clicking the link to the online assessment, participants were forwarded to a landing page that provided detailed information about the study. After providing informed consent, participants were asked to answer socio-demographic questions and provide information on music background, personality, and current mood.

Before hearing the first song, participants were instructed to set the volume to a comfortable level and were given the opportunity to familiarize themselves with all the GEMS terms. The order of terms varied randomly from participant to participant to avoid sequence effects, but was held constant across songs for any individual participant to facilitate orientation. Rating instructions specified that felt (rather than perceived) emotion be rated as follows:

The list of emotion terms below is used to describe feelings evoked by music. It captures feelings that are actually experienced by the listener (i.e., ‘I feel sad when listening to the music’). This in contrast to an emotional characterization of music or to the perceived emotional expression of the music (a funeral march may sound sad without necessarily making us feel sad).

Each music excerpt was presented twice. The first time, participants were instructed to merely listen to the music, whereas the second time they were asked to select emotion terms that matched their emotional experience. Participants were informed that not selecting an item meant that the respective feeling was not experienced. The selected terms were displayed in a separate space that asked participants to rate the intensity of the selected emotions by means of a slider on a scale of 0 to 100. Also included was an open text box for adding up to three further emotion terms that were not included in the GEMS. Before the emotion rating part, participants were asked to indicate familiarity with the song and liking of the song.

After rating a full set of 10–12 songs, participants were asked whether they wanted to rate an additional set or proceed to the final questionnaire page, which also asked participants about music usage, genre preferences, and their empathic traits (see SA). At the end of the assessment, participants received feedback on excerpts they had listened to, as well as a summary of their scores on some of the personality questionnaires.

## Results

### Interrater agreement and rater-to-interrater reliability ratios

On average, participants rated 18 music excerpts (*M* = 18.46, *SD* = 4.32, *Mdn* = 20, *Min* = 3, *Max* = 30). The exact distribution of number of raters across excerpts is plotted in Figure S1. To determine the interrater reliability in emotion ratings for the excerpts, we computed the ICC, which is one of the most widely used metrics to determine interrater agreement. The ICC was computed for agreement across GEMS items and across GEMS dimensions using the two-way random effects average measure model for consistency (McGraw & Wong, 1996). In accordance with the interpretations suggested by Koo and Li (2016), values between 0.50 and 0.75 can be considered to indicate moderate reliability, values between 0.75 and 0.90 good reliability, and values > 0.90 excellent reliability. Overall, the number of raters ranged from 11 to 101 (*M* = 28.76; *SD* = 7.99; *Mdn* = 28).

The critical importance of interrater reliability for determining the required number of annotators is best illustrated with an example. Assume that prior testing had shown annotator consensus to be perfect for a given music excerpt (ICC = 1.00). In this hypothetical scenario, a single annotator would be sufficient to determine the emotional effect of the excerpt, because any additional annotator would add only redundant information. The more interrater consensus deviates from perfect, the greater the number of annotators necessary to obtain a stable average value. A profile or pattern of emotion scores for a given music excerpt may be described as stable when ratings by additional annotators would leave the pattern of mean scores essentially unaltered.

**Fig. 2:**
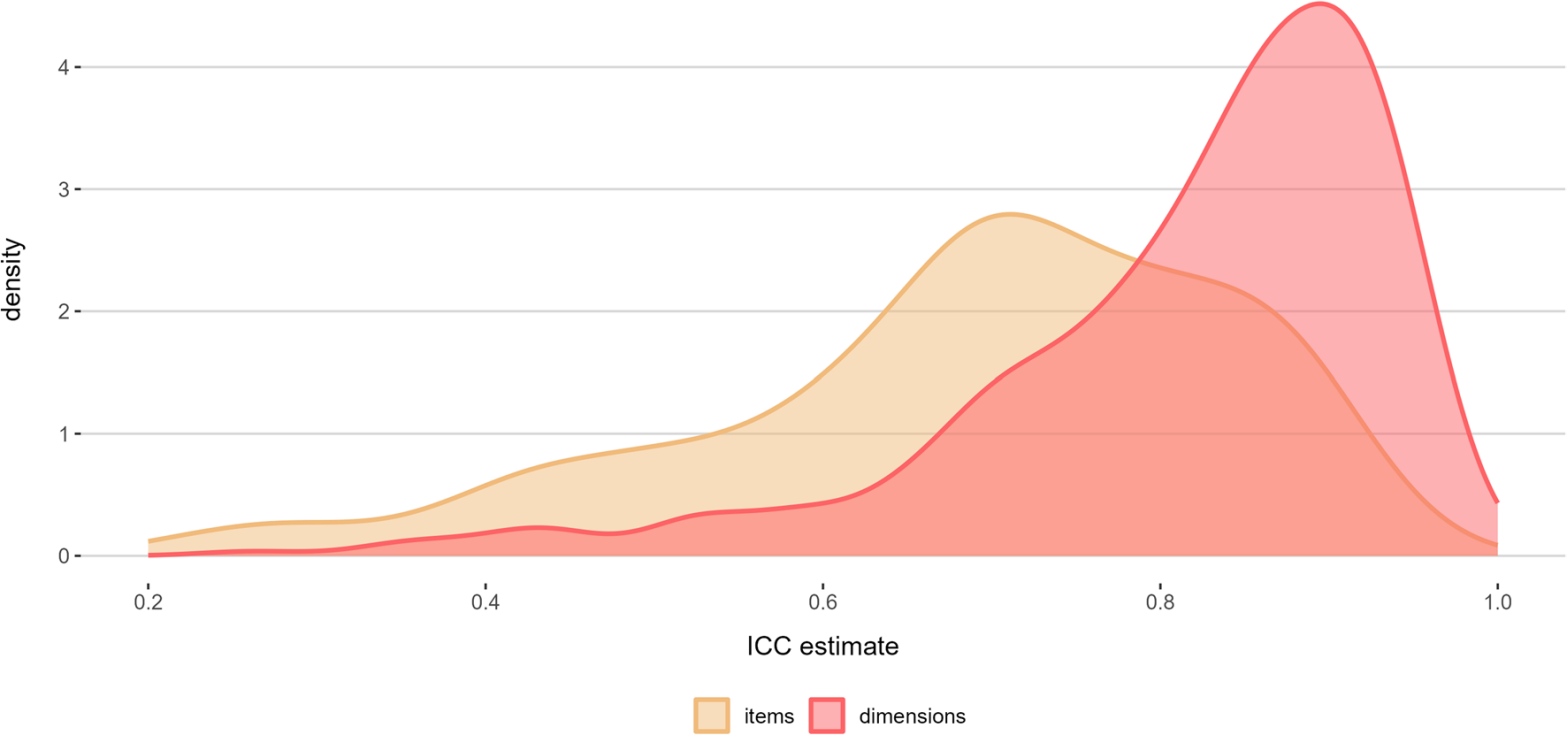
Density plot of ICC estimates for both item (yellow) and dimension level (red) Note. Values < .20 are not shown due to their low occurrence (< 2.0%)

Computed across all 45 items, ICCs were in the upper moderate range (*M* = .68, *SD* = .17, *Mdn* = .71, range = .05‒.95; 95% confidence interval [CI] lower limit ≥ .50 for 65.1% of all songs). Computed across the nine GEMS dimensions, ICCs showed good interrater agreement (*M* = .80, *SD* = .16, *Mdn* = .85, *range* = −.25‒.97; 95% CI lower limit ≥ .50 for 70.1% of all songs). Figure 2 shows the distribution of ICC estimates at the item and dimensional level. The specific ICC for any given song can be retrieved from our online database (see “The database” subsection).

Because the average intercorrelation between all raters was known for each excerpt, these empirical values can be inserted into the Spearman-Brown prophecy formula to estimate the reliability that would result for any number of raters (see Revelle & Condon, 2019; Vet et al., 2017). Table 1 illustrates the results of these computations. For example, to obtain a reliability of ≥ .50 in about 80% of music excerpts, 10 raters would have been sufficient; to obtain a reliability of ≥ .60 in 80% of music excerpts, the required number of annotators increases to 15 raters, and so forth (see Table 1, right half).

**Table 1:** ICC estimates for differently sized groups of raters as derived from the Spearman–Brown prophecy formula

				% of musical excerpts				
	*M*	*SD*	*Mdn*	ICC				
				≥ .50	≥ .60	≥ .70	≥ .80	≥ .90
5 raters	0.49	0.18	0.50	52.5	29.4	11.3	0.8	NA
10 raters	0.64	0.18	0.67	80.5	66.8	45.6	20.3	0.5
15 raters	0.71	0.18	0.75	90.4	80.5	64.6	36.0	6.3
20 raters	0.76	0.17	0.80	94.2	88.2	75.8	53.6	15.1
25 raters	0.79	0.17	0.84	95.6	92.3	82.7	63.7	25.5
30 raters	0.81	0.18	0.86	96.7	94.2	86.8	71.2	31.0
35 raters	0.83	0.19	0.88	96.7	95.3	90.4	76.9	41.2

Left side of table: average ICCs as a function of number of raters. Right side of table: percentage of music excerpts achieving set levels of interrater reliability given varying numbers of raters

We found that interrater consensus was associated with a number of factors. For example, the more a music piece was liked, the higher the resulting consensus (*r* = .25, *p* < .001). Furthermore, music evoking vitality emotions (joyful activation and energy) was positively associated with interrater consensus (*r* = .28, *p* < .001), and remained significant even after controlling for liking (*r* = .22, *p* < .001). Unease-evoking music was slightly negatively associated with consensus (*r* = −.12, *p* < .001), but this association was no longer significant after controlling for liking (*r* = −.02, *ns*). Looking at the extreme tails of the distribution, we found a disproportionate number of excerpts evoking the positively valenced vitality emotions among music pieces that were in the top 5% for interrater consensus. Conversely, there was a slightly elevated proportion of excerpts evoking the negatively valenced unease emotions among the bottom 5% for interrater consensus. Pop songs figured prominently in the top 5% category, whereas vocal music excerpts from the classical music genre were overrepresented in the bottom 5% category (see Table S2 for details).

### Relevance of GEMS emotion terms across genres

**Fig. 3:**
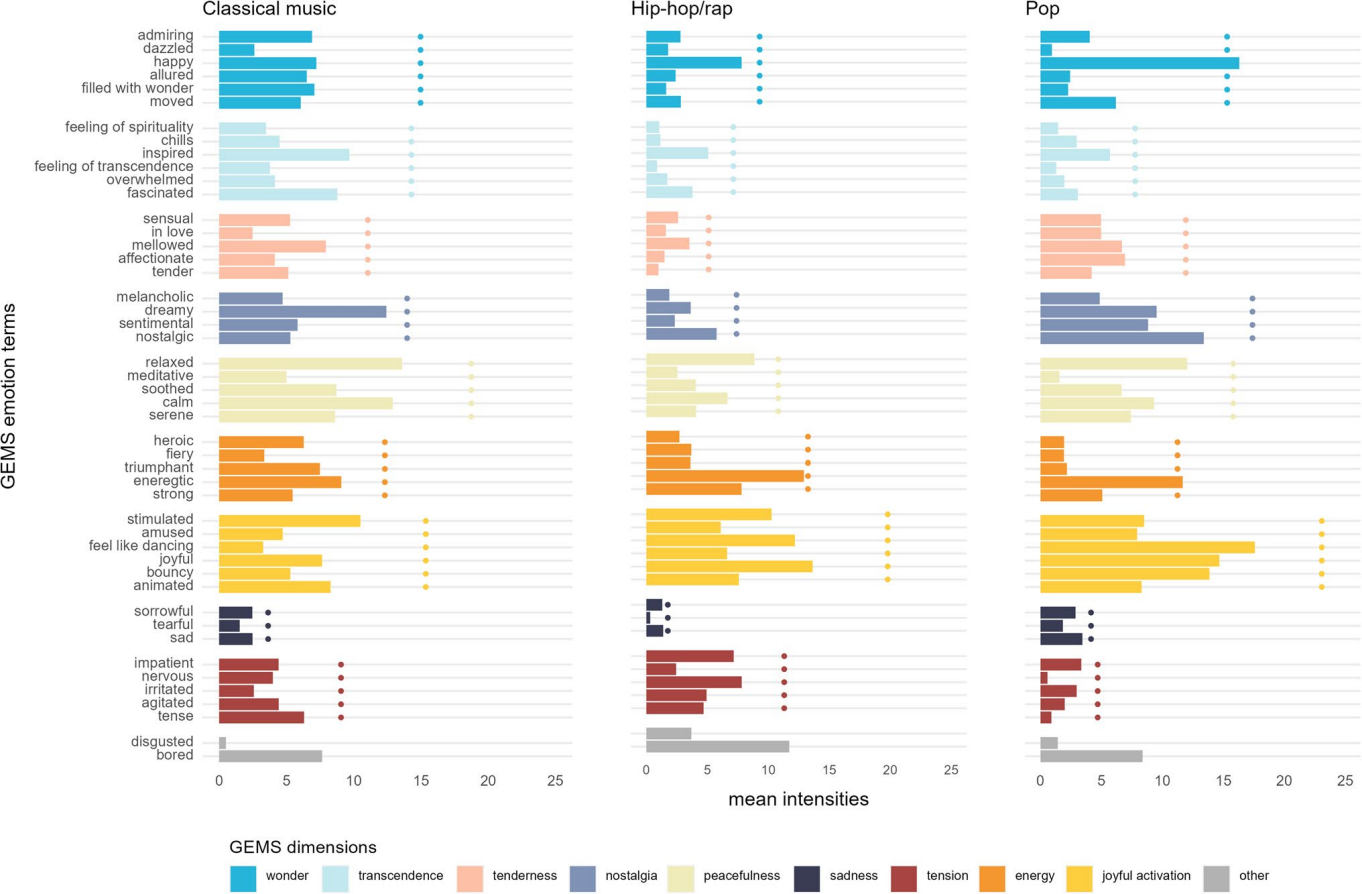
Mean intensities for individual items (bars) and dimensions (dotted lines) by genre

To evaluate the relevance of the GEMS terms, including bored and disgusted (see “Measures”), for describing emotions evoked across the three music genres, we computed the mean intensities of all items across all songs of a given genre, as is illustrated in Fig. 3. The average values of the nine dimensions ranged from 3.21 for sadness (*SD* = 4.88, *Mdn* = 1.39) to 19.76 for joyful activation (*SD* = 10.58, *Mdn* = 18.61). The values of the nine GEMS dimensions for individual songs ranged from 0.00 to 51.04, indicating that some songs had received a rating of 0 on some of the dimensions. Liking ratings for songs ranged from 1.74 to 4.36. The salience of items as derived from frequencies of selected GEMS terms was very similar to the intensity values (see Figure S2). Additional details on intensity ratings are shown in Table S3, whereas intercorrelations between dimensions and factors are shown in Table S4. Notably, ratings for the item “bored” were strongly inversely related to liking (*r* = −.47, *p* < .001), suggesting that both descriptors tap into one common disapproval factor 6.

**Fig. 4:**
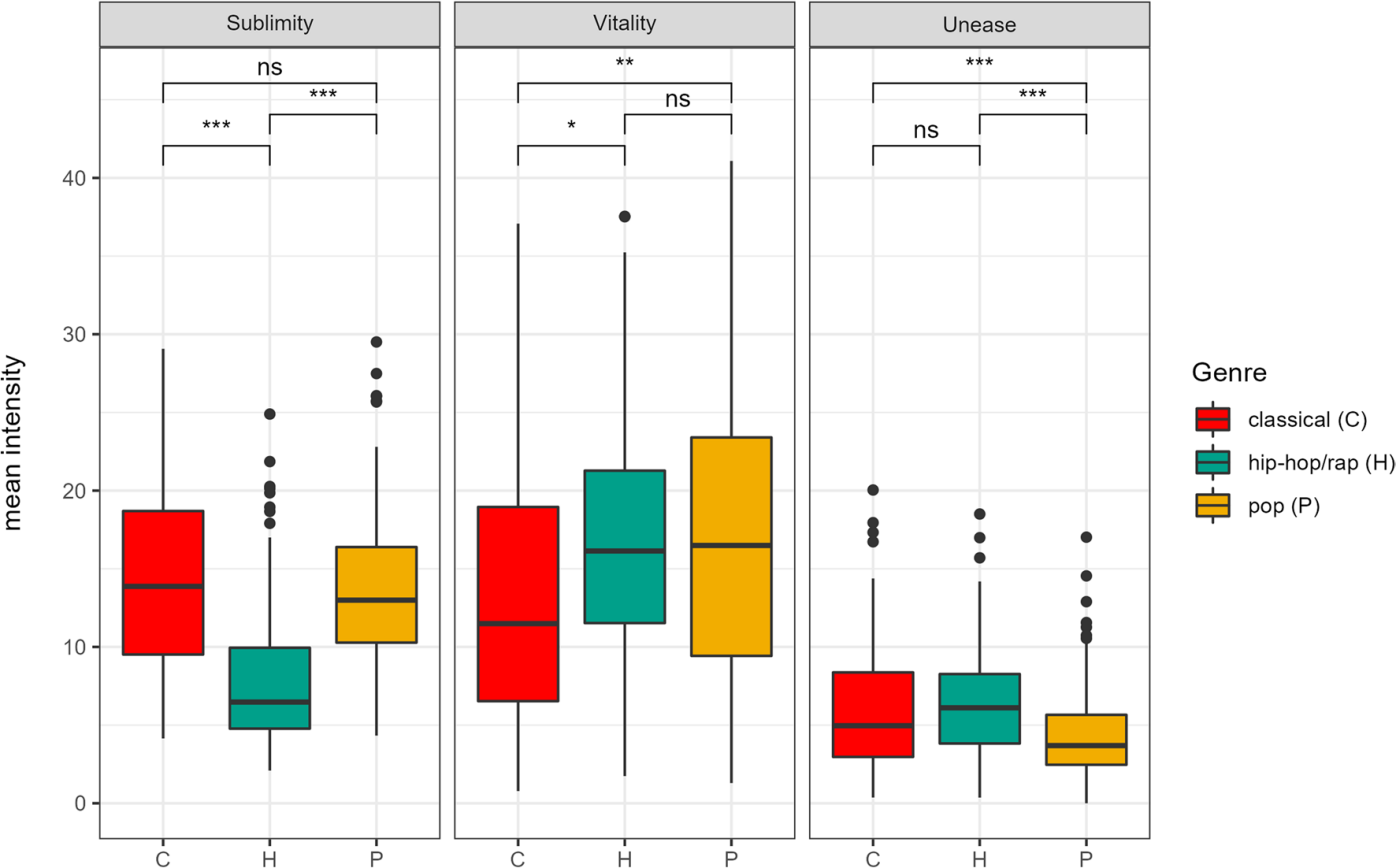
Boxplot graph of differences in factor mean levels by genre. *Note.* Post hoc tests using Tukey’s honest significant difference method indicated that participants experienced significantly lower sublimity in hip-hop/rap songs (M = 7.95, SD = 4.67) than they did in classical (M = 14.60, SD = 5.90) and pop music (M = 13.63, SD = 4.97). Conversely, they reported experiencing significantly higher levels of unease in hip-hop/rap (M = 6.53, SD = 3.51) and classical music (M = 6.35, SD = 4.30) than they did in pop music (M = 4.42, SD = 2.92). For vitality, levels were significantly lower in classical music (M = 13.84, SD = 8.97) than they were in pop (M = 17.15, SD = 9.33) and hip-hop/rap music (M = 16.52, SD = 7.89)

As can be gauged from Fig. 4, there were notable differences in music-evoked emotions across the three music genres. An analysis of variance on song-based means revealed that this was the case for all three emotion factors: sublimity, *F*(2,361) = 57.25, *p* < .001; vitality, *F*(2,361) = 4.60, *p* = .011; and unease, *F*(2,361) = 14.05, *p* < .001. The patterns are illustrated in Fig. 4, including the significance of pairwise comparisons (see Table S5 for the respective values at the dimension level).

The importance of the differential salience of terms across genres in the context of the present research lies primarily in indicating that all nine GEMS dimensions are indispensable for characterizing music-evoked emotion. For example, the category “tension” may seem of marginal utility for describing emotions evoked by pop music, but it has some importance for describing emotions evoked by classical music and by hip/hop. Similarly, the categories “tenderness” and “nostalgia” may seem dispensable for describing emotions evoked by hip/hop music, but this is not the case in relation to classical music and pop (see Fig. 3).

Because the present music selections cannot be seen as representative of the full spectrum of existing music, and because we used music excerpts rather than full versions of the songs or compositions, we attempted to obtain some estimate of generalizability. To this end, we compared the current frequencies with those obtained in another study in which live performances of classical, jazz, pop/rock, and world music were rated by 801 listeners, also using the GEMS (Zentner et al., 2008). Specifically, we correlated the GEMS item frequencies (i.e., percentages, see Figure S2) obtained in the current study with those obtained in the earlier study. The resulting association was sufficiently high (*r* = .75) to suggest that the relative salience of music-evoked emotions identified in the present study may be generalizable to some extent.

### Analysis of emotion terms added to the GEMS

As described in the Method section, after each excerpt, participants had the opportunity to add terms for any emotion they had felt but for which none of the GEMS terms seemed to provide a fitting label. We reasoned that if some emotion terms were to be consistently added, this would indicate a potential need for extending the GEMS. Conversely, if the terms added by the listeners were few and varied from listener to listener, this would support the content validity of the GEMS.

First, we identified terms that were not already included in the GEMS and were mentioned by at least 1% of our sample (i.e., ≥ 6 participants). This conservative criterion ensured that no potentially relevant terms would be left out, all while eliminating terms that were too idiosyncratic to represent the sentiment of a wider number of listeners. There were 25 such terms. Subsequently, we examined whether these 25 terms could be considered emotions by drawing from authoritative sources on the psychology of emotions (e.g., Ekman, 1992b; Oatley, 1999; Plutchik, 2001), especially those delineating differences between affect, mood, and emotion (e.g., Davidson et al., 1994; Ortony & Clore, 1989; Scherer, 2005). On the basis of these sources, all authors agreed that eight of the added terms referred to states that were not emotions, but rather affects, moods, physiological states, or general states of mind (e.g., *sleepy*, *tired*, *distracted*).

Finally, we examined whether each of the 17 remaining terms was actually different from the terms included in the GEMS. To this end, we checked each term against synonyms listed in the thesaurus of either the *Cambridge Dictionary* (dictionary.cambridge.org, 2022) or the *Merriam-Webster Dictionary* (merriam-webster.com/thesaurus, 2022). This analysis identified nine emotion terms that have at least one of the GEMS items listed as their synonym. This left eight emotion terms that were not included in the GEMS. The entire iterative process is summarized in Table 2 (an overview of all terms and the respective assignments is available upon request).

**Table 2:** Terms added to the GEMS by participants

Term	*n*	Not an emotion	GEMS synonym
sleepy	11	x	
thoughtful	10	x	
tired	10	x	
desire to sing	10	x	
cool	10	x	
indifferent	8	x	
distracted	7	x	
neutral	6	x	
annoyed	57		bored^b^, irritated^a,c^,
chilled	10		mellowed^c^, relaxed^c^
angry	9		disgusted^b^, irritated^b,c^
anxious	8		agitated^b^, impatient^a^, nervous^a^, tense^a^
peaceful	8		calm^a^, relaxed^b^, serene^a^
interested	7		allured^b^, fascinated^c^, stimulated^a^
intrigued	7		allured^b^, stimulated^a^
restless	6		bored^c,^ impatient^b^, nervous^b^, tense^b^
stressed	6		irritated^b,d^, nervous^b,d^, sorrowful^b,d^, tense^b,d^
confused	24		(dazzled^b^)
focused	9		*-*
aggressive	8		*-*
curious	8		-
confident	6		
motivated	6		-
surprised	6		(overwhelmed^a^)
uncomfortable	6		-

*n* refers to the number of different participants who mentioned the term. ^a^*Merriam-Webster* (first order); ^b^*Merriam-Webster* (second order); ^c^*Cambridge* (first order); ^d^actual synonym is *distressed*. Terms that meet the formal synonym criteria but did not appear fully equivalent semantically are placed in parentheses

Overall, the findings of the free-response analyses suggest that the GEMS does not leave out highly significant terms for describing music-evoked emotion. This does not imply that the GEMS provides an exhaustive mapping of music-evoked emotions, of course. Terms such as “curious” and “focused”, combined with “surprised”, appear to point to an attention/interest dimension that is not specifically represented in the GEMS and should be kept in mind for future studies.

### Effect of personal characteristics on music-evoked emotions

We also explored the impact of rater characteristics on ratings of music-evoked emotion. To this end, we ran multilevel regressions predicting intensity of music-evoked emotions by such factors as age, gender, personality traits, and music preferences, but also excerpt liking, excerpt familiarity, and current mood state (see SA). In line with previous findings (e.g., Aljanaki et al., 2016), the analyses showed excerpt liking to be the strongest and most consistent predictor of the intensity of emotions across music genres, followed by current mood and familiarity (see Table SA1). We also found that listeners experienced more intense positive emotions and less intense negative emotions when the excerpts matched their music preferences (see Table SA2). Other listener characteristics exhibited only small associations with music-evoked emotions regardless of genre. This may seem somewhat surprising, but is consistent with the relatively high interrater agreement found for a majority of music excerpts.

## The database

**Fig. 5:**
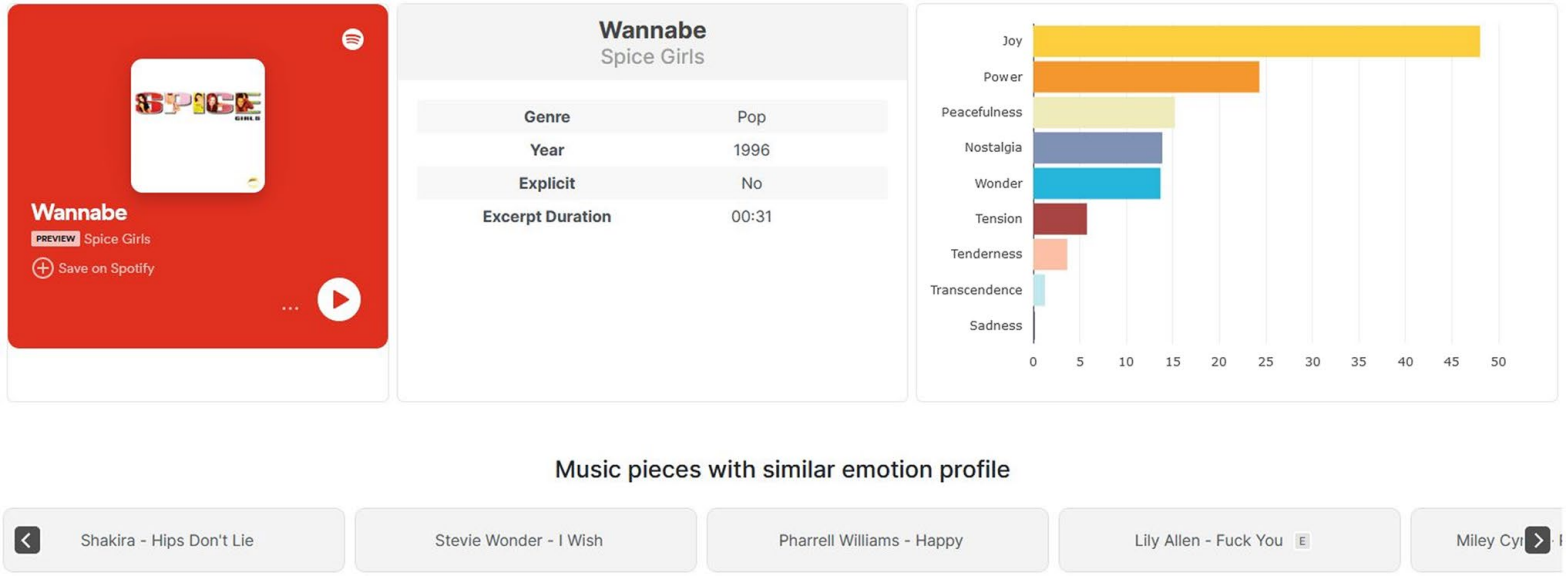
Screenshot of the database webpage Song Profile

In what follows, we provide a brief summary of the main functionalities of the database. For its capacity to let users find music excerpts that evoke specific emotions across various musical genres, we named it the Emotion-to-Music Mapping Atlas (EMMA). Although the term “atlas” may appear to overstate the scope of the database in its current stage, EMMA will be continuously expanded by new excerpts, new music genres, and additional functionalities. EMMA, which can be accessed online via psychologie-shiny.uibk.ac.at, is characterized by the following main features:

1. Extensive emotion ratings are provided for each excerpt and include average values for each of the nine GEMS dimensions and the three GEMS superordinate factors, as well as average ratings for liking and familiarity of the respective excerpts.
2. Interrater reliability (ICC) is provided for each excerpt so that researchers can decide for themselves whether or not the extent of agreement is sufficient for their purposes.
3. Excerpts can be filtered by various criteria (e.g., by genre, vocal/instrumental music, language) and sorted in ascending or descending order of intensity for any given emotion, as well as for interrater reliability, average familiarity, and liking. An open search box allows users to find excerpts based on title or artist.
4. For any selected excerpt, the user is offered recommendations for excerpts that share a similar emotion profile (see Fig. 5, bottom row). Similarity was computed from intercorrelations across all excerpts by using emotion ratings on the nine dimensions and the three factors. The 10 excerpts with highest similarity coefficients are displayed to the user. Apart from assisting the user in exploring the musical universe by way of emotion, researchers may benefit from this functionality in searching for music excerpts that have similar emotional effects but differ with regard to other features such as genre, period, or instrumentation.
5. Data can be displayed at various levels of detail: a “basic level”, which has all the songs with their respective emotion ratings; an “expert level”, which includes additional variables, such as ratings of liking, familiarity, and ICCs, as well as links to the respective YouTube clips with the start and end times of the examined excerpts; and, finally, a “custom level” that allows users to select a bespoke set of variables.
6. Data can be directly downloaded as a csv file at any of the above-mentioned levels of detail.

## Discussion

Music databases including annotations for emotion have grown in recent years, but the benefits of this development are tempered by several factors. For one, the reliability of the annotations is often unknown, and the requirements for obtaining reliable annotations have received relatively little attention. Second, the emotion concepts that are most frequently used for annotating or predicting musical emotions lack either granularity (affective circumplex) or pertinence to musically evoked emotions (basic emotions). Third, emotion tags or measures are mostly used to characterize perceived emotion. As such, they may not do justice to the uniqueness of music-induced emotions, potentially making annotations equivocal or inexact. Finally, the datasets are seldom set up in a way that is readily accessible and easy to use. In the current study, we proposed a number of research steps that can help redress some of these issues. On this basis, we initiated a new online database that differs from previous ones in several respects. In what follows, we discuss some of the most important findings and their implications for music and emotion databases.

### Required number of raters for reliable annotations

As the first of their kind, our rater-to-reliability analyses provide important insights as to how resource effectiveness in the use of human annotators may be maximized. Our analyses suggest that obtaining stable estimates of an excerpt’s emotional effect requires a minimum of 10 annotators in most cases. In turn, the incremental value of using more than 25 raters is questionable. In terms of resource efficiency, the optimal number likely sits between 10 and 20 annotators, but it should be clear that this is only a broad estimate.

Indeed, we identified factors that tend to boost or attenuate interrater reliability. For example, consistent with the results by Alijanaki et al. (2016), liking of the music tended to increase interrater agreement. Here, we showed that interrater agreement is also higher in music excerpts that induce positive emotions. These findings are helpful in indicating when a number of raters in the lower bound of the recommended range may be sufficient to attain adequate interrater reliability, and when it is advisable to aim for a number in the upper end. As a note of caution, we should add that the importance of attaining high interrater agreement depends on the aims of a particular study. For example, a very high degree of agreement is bound to attenuate the effect of individual differences on music evoked-emotion. Thus, researchers interested in studying the impact of individual differences on music-evoked emotions will fare better by selecting excerpts that evoke a certain degree of variability in emotional responses.

Our supplementary analyses of specific person characteristics, though not a focus of this research, may also be taken profitably into account when deciding on the music excerpts to use for particular purposes. The single strongest predictor of intensity of experienced emotion was listeners’ liking of an excerpt, which is consistent with the literature (Aljanaki et al., 2016). When liking for specific excerpts cannot be known prior to a study, our supplementary analyses suggest that music preferences may serve as rough proxy, insofar as excerpts matching listeners’ preferred music genres tended to evoke more intense positive emotions and less intense negative emotions than excerpts that deviated from listeners’ preferred music genres.

### Efficiency and content validity of the GEMS

A second goal of the study was to broaden the empirical basis for evaluating the GEMS as a system for emotion annotation and classification in music databases and in music information retrieval more generally. In regard to efficiency, our results indicate that all nine categories were relevant in one genre or another, with sadness and tension being the least prominent categories. This is not surprising, because although music can be perceived as being expressive of negative emotions, it rarely induces such emotions in the listener. Within a given category, not all terms were used with equal frequency. This finding indicates that the short version of the GEMS, the GEMS-25, might provide a more efficient alternative to the GEMS-45 when time is critical.

Whereas including too many terms hampers efficiency, leaving out terms that listeners consider important for describing music-evoked emotions would compromise validity. We found that few terms were consistently added by participants to those already included in the GEMS. In essence, the additions indicate that a cluster of emotions defined by interest, curiosity, and attention may not be sufficiently represented in the GEMS, and that that the term “annoyed” would represent a useful addition, perhaps to be subsumed under the “tension” category. Overall, however, the GEMS appears to have sufficient breadth of emotional coverage to accommodate a variety of popular musical genres. In further support of this conclusion, it is worth noting that the frequencies of GEMS terms usage found for the present music selections were consistent with the frequencies of GEMS terms used for describing emotions evoked by other types of music (Juslin et al., 2008; Zentner et al., 2008).

Although the generalizability of the current findings to non-Western populations remains to be determined, it is worth noting that a cross-cultural investigation of respondents from individualist and collectivist cultures found patterns in the prevalence of experienced emotions that are relatively similar to those identified in the present study emotions (Juslin et al., 2016). It is also worth noting that, overall, our findings related to genres are consistent with the literature. For example, classical music was found to elicit predominantly emotions of sublimity, such as tenderness and wonder, whereas pop music showed high levels of joy and nostalgia (see Aljanaki et al., 2016; Juslin et al., 2022; Zentner et al., 2008). Also in line with the literature (Aljanaki et al., 2016; Juslin et al. 2022), we found classical and pop music to evoke similar levels of sadness.

### Developing and expanding EMMA

Addressing issues of interrater agreement and taxonomic adequacy was essential for developing a database of music excerpts that is systematic in its approach to emotion annotation and that may serve as a guide for the development of similar databases. In the organization of our database, we prioritized (a) ease of access by making the database available on a dedicated website, (b) user-friendliness by providing transparent organization, and (c) flexibility by including a variety of easily retrievable search and filtering functions. It should be clear that, although going a step beyond currently existing databases, EMMA is a work in progress and it will be continuously expanded by new music excerpts and functionalities. As an example, our most recent project added several new music (sub)genres to the collection described in the present article, including Disco, Funk, Jazz, Metal, Punk, Rock, Soul, Swing, and Trance. We expect the GEMS ratings for over 450 music excerpts from these genres to be incorporated into EMMA in the course of 2024.

### Limitations

The present contribution should be evaluated in the context of several limitations. First, by including Western art music, pop, and hip-hop/rap, we have tried go beyond stimuli used in previous studies (Warrenburg, 2021) and better reflect the listening habits of a large part of current Western populations. Even so, the selections are by no means representative of the variety of existing musical styles and cultures. Future investigations should assess the reproducibility of these findings in listeners from more diverse ethnic and cultural contexts.

Second, the current database is one of the few whose music excerpts were rated for felt rather than perceived emotion, and there is evidence suggesting that GEMS ratings do capture felt emotions as noted in the Introduction. Even so, the separation between perceived and felt emotion is not always straightforward (Schubert, 2013). Thus, Juslin and Västfjäll (2008) suggested that emotional contagion might lead to certain emotions being induced by a music piece as the listener “mimics” its perceived emotional expression. There is also the possibility that when repeatedly experiencing little or no emotion, some of our listeners may have preferred to report perceived emotions rather than no emotion at all in accordance with the good-subject effect (Nichols & Maner, 2008; Rosnow & Rosenthal, 1997).

Third, the extent to which lyrics influenced participants’ emotional states remains unclear. Previous research has indicated that sad lyrics, in particular, enhance listeners’ experience of sadness in music, whereas no such effects were found for happy lyrics (Barradas & Sakka, 2022; Brattico & Pearce, 2013). Hence, in the current study, feelings of nostalgia, in particular in pop and hip-hop/rap excerpts, might have been influenced by the lyrics; however, this hypothesis needs to be examined in future studies.

Fourth, because EMMA aims to reflect music listening in naturalistic contexts, and because music predominantly elicits positive emotions in naturalistic listening environments (Juslin et al., 2022), the number of excerpts for negative emotion induction in EMMA is rather limited at present. In expanding EMMA with music new subgenres, such as film or gaming music, we expect that the choice of excerpts for negative emotion induction will gradually increase.

### Applications and outlook

EMMA may prove to be of value to scholars from various disciplines. For example, the database can assist researchers in finding stimuli for the induction of specific affective states in experimental settings. In contrast to most existing databases, EMMA offers a detailed portrait of the emotional effects of music excerpts rather than a sketch based on dimensional ratings for valence and arousal. In addition, average excerpt liking and familiarity are reported, as well as interrater reliability, so that researchers can base their selection of stimuli on an extensive range of metrics. For example, users can choose excerpts that are less likely to be known by participants in order to reduce the influence of stimulus familiarity, or choose stimuli that are likely to induce a homogeneous or heterogeneous emotional effect based on high or low interrater agreement.

The database could also benefit machine learning approaches because of the importance of high-quality human annotations for validating the results generated by music recommender systems (Emam et al., 2021). In comparison with previous databases, the current one includes emotionally richer annotations that could provide a point of departure for developing more powerful machine learning approaches to prediction and classification, notably in the context of MER (see, Gómez-Cañón et al., 2021; Turchet et al., 2023; Won et al., 2021). Future studies could also run audio feature extraction software, such as MIRtoolbox (Lartillot et al., 2008), Essentia (Bogdanov et al., 2013), or openSMILE (Eyben et al., 2010), on EMMA excerpts so as to identify acoustic and musical features associated with music-evoked emotions.

Progress along these lines could pave the way for improvements in practical applications, such as improved approaches to categorization of music collections, improved music recommender systems that leverage nuanced emotion information for optimizing personalization, or the development of generative models for the creation of emotion-specific music. It may also be useful in a wide range of psychological applications by assisting practitioners in finding music with an emotional profile that fits the needs of clients. More generally, the approach advanced here may serve as a framework for the development of future databases dedicated to music and emotion.

### Supplementary Information

Below is the link to the electronic supplementary material.

Supplementary file1 (DOCX 162 KB)

Supplementary file2 (DOCX 297 KB)

## Data Availability

The datasets generated during and analysed during the current study are available in the Open Science Framework (OSF) repository, https://osf.io/7ptmd/. In addition, data on emotion profiles are available at the interactive database webpage https://psychologie-shiny.uibk.ac.at/.

## Footnotes

1 http://www.projects.science.uu.nl/memotion/emotifydata/

2 https://github.com/HuiZhangDB/PMEmo

3 The original denomination of this dimension is “power” (Zentner et al., 2008). However, because of the ambiguous meaning of “power” when translated into some other languages, including German (“Macht”), we use the less equivocal “energy” instead.

4 Relative to the formula published in Gerstgrasser et al. (2022), the abbreviation for the number of selected emotions, previously denoted as “xs”, was changed to “s” for clarity.

5 Because distributions and internal consistencies for the English and German language versions of the measures were relatively similar, we report values for the whole sample.

6 Before computing mean intensities and frequencies of items, dimensions, and factors, we tested whether emotion ratings were affected by the position of the songs within the sets of songs, but found such effects to be minimal (see section “Order of Songs and Emotions Experienced” at the end of the Supplemental Materials).
