# Supplementary material for: The Emotion-to-Music Mapping Atlas (EMMA): A systematically organized online database of emotionally evocative music excerpts
Source: Behav Res Methods. 2024 Jan 30;56(4):3560–77. doi: 10.3758/s13428-024-02336-0 (PMC11133078; doi:10.3758/s13428-024-02336-0)
Supplement: Supplementary file 1 — Supplementary file1 (DOCX 162 KB) [file 13428_2024_2336_MOESM1_ESM.docx]

## “The Emotion-to-Music Mapping Atlas (EMMA): A Systematically Organized Online Database of Emotionally Evocative Music Excerpts”

## Supplementary Appendix (SA):

## **Rater Characteristics and Their Effect on Music-Evoked Emotion**

### We assessed a series of personal characteristics of the raters (see Measures below) and examined their effect on ratings of music-evoked emotion (see Results below). To this end, we ran nine multilevel models, one model for each higher order emotion factor in each of the three genres. All listener features were entered simultaneously as predictors. Prior to entering them in the model, all variables were standardized. Because every participant heard multiple songs per genre, we calculated the models with a random intercept of participant ID and a random intercept of musical piece. The results are presented in Table SA1. Because excerpt liking, excerpt familiarity, and mood state are seldom known prior to a study, they cannot serve as criteria to select music excerpts in advance. However, since individuals’ preferred style of music is by definition liked and familiar, we reasoned that genre preferences could act as a passable proxy for excerpt liking and familiarity. To examine this assumption, we ran the same type of multilevel analyses but omitted the variables “excerpt liking” and “excerpt familiarity”. As shown in Table SA2, listeners experienced more positive emotions (Sublimity, Vitality) and less negative emotions (Unease) when the excerpts matched their music preferences.

### Measures

***Music Background***

Information on participants’ musical background was assessed with multiple indicators: (a) self-rated level of musicianship (0 = *non-musician*; 1 = *music-loving non-musician*; 2 = *amateur musician*; 3 = *semi-professional musician*; 4 = *professional musician*); (b) music qualification (0 = *no qualification*; 1 = *non-academic music qualification or awards*; 2 = *currently enrolled in academic music studies*; 3 = *graduated in academic music studies*); and (c) music listening frequency (“How often do you listen to music?”; 0 = *Never*, 1 = *Sometimes*; 2 = *1-2 days a week*; 3 = *3-4 days a week*; 4 = *5-6 days a week*; 5 = *every day*). The three components were internally consistent (*ω* = .67) and were therefore combined into one composite score termed “music background.”

***NEO-Five-Factor Inventory***

Personality traits Openness for Experience, Extraversion, and Neuroticism were assessed by using a short version of the NEO-Five-Factor Inventory (NEO-FFI-30; Körner et al., 2008). The NEO-FFI-30 is a short version of the German translation of the NEO-FFI (Borkenau & Ostendorf, 1993) and comprises six items per subscale. In the English version, the respective items were drawn from the English version of the NEO-FFI by Costa and McCrae (Borkenau & Ostendorf, 1993). Internal consistency (*ω*) was .86 for Openness for Experience, .86 for Extraversion, and .90 for Neuroticism.

***Positive and Negative Affect Schedule***

Participants’ mood was assessed before and after the music rating with a short form of the Positive and Negative Affect Schedule (I-PANAS-SF; Thompson, 2007). The German translation of the 10-item questionnaire was drawn from Breyer and Bluemke (2016). Participants were asked to rate their current mood on a 5-point scale (0 = *not at all* to 4 = *extremely*). The I-PANAS-SF has shown to be a valid and reliable measure of positive (PA) and negative affect (NA). Internal consistency was found to range from *ω =*.83–.86 across type of affect (positive and negative) and assessment times (before and after music rating).

***Uses of Music Inventory***

Participants’ motives for listening to music were assessed by the Uses of Music Inventory (Chamorro-Premuzic & Furnham, 2007). The Uses of Music Inventory includes three dimensions, each comprising five items: (1) *Emotional Use* (e.g., ‘Listening to music really affects my mood’), (2) *Cognitive Use* (e.g., ‘I often enjoy analysing complex musical compositions’), and (3) *Background Use* (e.g., ‘I enjoy listening to music while I work’), to be rated on a 5-point scale ranging from *strongly disagree* to *strongly agree*. Items were translated into German and back-translated by three independent experts. In the current sample, internal consistencies were *ω =*.63 for Emotional Use, *ω =*.74 for Cognitive Use, and *ω =*.75 for Background Use.

***Short Test of Music Preferences***

The Short Test of Music Preferences (STOMP; Rentfrow & Gosling, 2003) assesses participants’ basic preference levels for 14 genres on a 7-point scale ranging from *strongly dislike* to *strongly like*. Items were translated into German by two independent experts. In the current analyses, we used scores of participants’ preferences for the genres represented in the music stimuli (i.e., Classical, Hip-Hop/Rap, and Pop), as well as the four higher level music preference dimensions: Reflective & Complex (*ω* = .70); Intense & Rebellious (*ω* = .57); Upbeat & Conventional (*ω* = .55); Energetic & Rhythmic (*ω* = .60).

***Interpersonal Reactivity Index***

A short version of the German Interpersonal Reactivity Index (IRI-S D; Paulus, 2009) was used to assess participants’ empathic traits. The 12 items were answered on a 5-point rating scale ranging from *never* to *always*. English wording of the respective items was drawn from the original version of the IRI by Davis (1980). Internal consistency (*ω*) of the overall empathy score was .87.

### Table SA1

Multilevel Regression Results of Listener Features Predicting Overall Intensities in Emotion Factors Sublimity, Vitality, and Unease

|  | **Music Stimulus Type** | | | | | | |  |  | | |
| --- | --- | --- | --- | --- | --- | --- | --- | --- | --- | --- | --- |
|  | **Classical (C)** | | |  | **Hip-hop/Rap (HH)** | | |  | **Pop (P)** | | |
|  | **Sub** | **Vit** | **Une** |  | **Sub** | **Vit** | **Une** |  | **Sub** | **Vit** | **Une** |
| Gender:female | .01 | .00 | .03 |  | -.02 | .02 | .01 |  | -.05 | .01 | .01 |
| Age | -.06 | -.04 | -.10** |  | .00 | -.05 | -.03 |  | -.05 | -.05* | -.04 |
| Highest level of education | -.01 | .01 | -.02 |  | .02 | .01 | -.02 |  | -.02 | .00 | -.01 |
| Excerpt familiarity | .00 | .07** | .01 |  | .00 | .07** | .05** |  | .04* | .08** | .12** |
| Excerpt liking | .44** | .29** | -.24** |  | .43** | .49** | -.43** |  | .44** | .37** | -.26** |
| Music background | .01 | .00 | .03 |  | .00 | .02 | .02 |  | .01 | .04 | .00 |
| UMI Emotional regulation | .04 | .03 | .03 |  | .04 | .02 | .05 |  | .03 | .00 | .06* |
| UMI Cognitive elaboration | -.01 | -.02 | .00 |  | .05 | -.01 | .01 |  | .00 | -.03 | .02 |
| UMI Background | .00 | .02 | -.02 |  | .02 | .06* | -.02 |  | .01 | .02 | .01 |
| NEO-FFI Openness | -.01 | .01 | .10** |  | -.05 | .02 | .04 |  | .00 | .00 | .06* |
| NEO-FFI Extraversion | .04 | .02 | .07* |  | .04 | .03 | .01 |  | .04 | .03 | .00 |
| NEO-FFI Neuroticism | .00 | .04 | .03 |  | .00 | .02 | -.01 |  | .04 | .02 | .00 |
| IRI Empathy | .10** | .03 | -.02 |  | .10** | .01 | .02 |  | .07* | .03 | .03 |
| PANAS positive mood | .05 | .03 | -.01 |  | .07** | .06* | .00 |  | .11** | .05* | .02 |
| PANAS negative mood | -.06* | -.02 | .06* |  | -.01 | -.05 | .05* |  | -.05 | -.03 | .04 |
| STOMP: Classical | .03 | .01 | .01 |  | -.02 | -.02 | .02 |  | .01 | .00 | -.02 |
| STOMP: Hip/Hop | -.02 | -.02 | -.03 |  | .00 | .01 | -.07** |  | .00 | .00 | -.01 |
| STOMP: Pop | -.01 | .00 | .03 |  | -.02 | -.02 | .01 |  | .01 | .01 | .02 |
| Marginal R^2^ / Conditional R^2^ | .26 / .59 | .12 / .52 | .07 / .32 |  | .25 / .55 | .31 / .60 | .19 / .38 |  | .27 / .58 | .20 / .52 | .06 / .24 |

*Note*. Sub = GEMS Sublimity; Vit = GEMS Vitality; Une = GEMS Unease; UMI = Uses of Music Inventory; NEO-FFI = NEO-Five-Factor Inventory; IRI = Interpersonal Reactivity Index; PANAS = Positive and Negative Affect Schedule; STOMP = Short Test of Music Preferences. Values are *β* coefficients.

*p* < .05 * *p* < .01**

### Table SA2

Multilevel Regression Results after Removal of “Excerpt Liking” and “Excerpt Familiarity” ^a^

|  | **Classical (C)** | | |  | **Hip-hop/Rap (H)** | | |  | **Pop (P)** | | |
| --- | --- | --- | --- | --- | --- | --- | --- | --- | --- | --- | --- |
|  | **Sub** | **Vit** | **Une** |  | **Sub** | **Vit** | **Une** |  | **Sub** | **Vit** | **Une** |
| STOMP: Classical | .28** | .20** | -.13** |  | -.03 | -.03 | .03 |  | < .01 | -.01 | -.05 |
| STOMP: Hip/Hop | -.06 | -.05 | -.03 |  | .24** | .30** | -.35** |  | .01 | .01 | -.03 |
| STOMP: Pop | .02 | .04 | .03 |  | -.05 | -.05 | .04 |  | .10* | .11* | -.01 |

*Note*. Sub = GEMS Sublimity; Vit = GEMS Vitality; Une = GEMS Unease; STOMP = Short Test of Music Preferences. Values are *β* coefficients.

*p* < .05 * *p* < .01**

*^a^* Effects of other predictors listed in Table SA1 remained essentially unchanged*.*

**References**

Borkenau, P., & Ostendorf, F. (1993). *NEO-Fünf-Faktoren-Inventar (NEO-FFI) nach Costa und McCrae: Handanweisung* [NEO Five-Factor Inventory (NEO-FFI) by Costa and McCrae: Manual]. Hogrefe, Verl. für Psychologie.

Breyer, B., & Bluemke, M. (2016). *Deutsche Version der Positive and Negative Affect Schedule PANAS (GESIS Panel)* [German version of the Positive and Negative Affect Schedule PANAS (GESIS Panel)]. https://doi.org/10.6102/ZIS242

Chamorro-Premuzic, T., & Furnham, A. (2007). Personality and music: Can traits explain how people use music in everyday life? *British Journal of Psychology*, *98*(2), 175–185. https://doi.org/10.1348/000712606X111177

Davis, M. H. (1980). *A multidimensional approach to individual differences in empathy*. *Catalog of selected documents in psychology : [Microfiche collection of full-text documents]: 2124 = vol. 10,4*. American Psycholog. Ass., Journal Suppl. Abstract Service.

Körner, A., Geyer, M., Roth, M., Drapeau, M., Schmutzer, G., Albani, C., Schumann, S., & Brähler, E. (2008). Persönlichkeitsdiagnostik mit dem NEO-Fünf-Faktoren-Inventar: Die 30-Item-Kurzversion (NEO-FFI-30) [Personality assessment with the NEO-Five-Factor Inventory: the 30-Item-Short-Version (NEO-FFI-30)]. *PPmP - Psychotherapie · Psychosomatik · Medizinische Psychologie*, *58*(6), 238–245. https://doi.org/10.1055/s-2007-986199

Paulus, C. (2009). *Der Saarbrücker Persönlichkeitsfragebogen SPF(IRI) zur Messung von Empathie: Psychometrische Evaluation der deutschen Version des Interpersonal Reactivity Index* [The Saarbrueck Personality Questionnaire on Empathy: Psychometric evaluation of the German version of the Interpersonal Reactivity Index]. https://doi.org/10.23668/psycharchives.9249

Rentfrow, P. J., & Gosling, S. D. (2003). The do re mi's of everyday life: The structure and personality correlates of music preferences. *Journal of Personality and Social Psychology*, *84*(6), 1236–1256. https://doi.org/10.1037/0022-3514.84.6.1236

Thompson, E. R. (2007). Development and validation of an internationally reliable short-form of the Positive and Negative Affect Schedule (PANAS). *Journal of Cross-Cultural Psychology*, *38*(2), 227–242. https://doi.org/10.1177/0022022106297301
