# Supplementary material for: The Emotion-to-Music Mapping Atlas (EMMA): A systematically organized online database of emotionally evocative music excerpts
Source: Behav Res Methods. 2024 Jan 30;56(4):3560–77. doi: 10.3758/s13428-024-02336-0 (PMC11133078; doi:10.3758/s13428-024-02336-0)
Supplement: Supplementary file 2 — Supplementary file2 (DOCX 297 KB) [file 13428_2024_2336_MOESM2_ESM.docx]

## Supplemental Online Materials for

## “The Emotion-to-Music Mapping Atlas (EMMA): A Systematically Organized Online Database of Emotionally Evocative Music Excerpts”

### Table S1 *Descriptive Statistics for Socio-Demographic Data for the Total Sample and Across Language Versions*

|  | *Total* | |  | *English* | |  | *German* | |
| --- | --- | --- | --- | --- | --- | --- | --- | --- |
|  | *n* | *%* |  | *n* | *%* |  | *n* | *%* |
| **Assessment language** |  |  |  |  |  |  |  |  |
| English | 306 | 54.0 |  |  |  |  |  |  |
| German | 258 | 45.5 |  |  |  |  |  |  |
| NA | 3 | 0.5 |  |  |  |  |  |  |
| **Skills in assessment language** |  |  |  |  |  |  |  |  |
| Beginner | 5 | 0.9 |  | 1 | 0.3 |  | 4 | 1.6 |
| Intermediate | 10 | 1.8 |  | 9 | 2.9 |  | 1 | 0.4 |
| Advanced | 37 | 6.5 |  | 28 | 9.2 |  | 9 | 3.5 |
| Mother tongue | 512 | 90.3 |  | 268 | 87.6 |  | 224 | 94.6 |
| NA | 3 | 0.5 |  |  |  |  |  |  |
| **Sex** |  |  |  |  |  |  |  |  |
| Male | 243 | 42.9 |  | 143 | 46.7 |  | 100 | 38.8 |
| Female | 316 | 55.7 |  | 159 | 52.0 |  | 157 | 60.9 |
| Other | 5 | 0.9 |  | 4 | 1.3 |  | 1 | 0.4 |
| NA | 3 | 0.5 |  |  |  |  |  |  |
| **Nationality** |  |  |  |  |  |  |  |  |
| Australia | 10 | 1.8 |  | 10 | 3.3 |  | 0 | 0.0 |
| Austria | 47 | 8.3 |  | 1 | 0.3 |  | 46 | 17.8 |
| Canada | 9 | 1.6 |  | 9 | 2.9 |  | 0 | 0.0 |
| Germany | 180 | 31.7 |  | 1 | 0.3 |  | 179 | 69.4 |
| Greece | 2 | 0.4 |  | 2 | 0.7 |  | 0 | 0.0 |
| Italy | 25 | 4.4 |  | 2 | 0.7 |  | 23 | 8.9 |
| Latvia | 2 | 0.4 |  | 1 | 0.3 |  | 1 | 0.4 |
| Malysia | 3 | 0.5 |  | 3 | 1.0 |  | 0 | 0.0 |
| New Zealand | 3 | 0.5 |  | 3 | 1.0 |  | 0 | 0.0 |
| South Africa | 13 | 2.3 |  | 13 | 4.2 |  | 0 | 0.0 |
| United Kingdom or Republic of Ireland | 121 | 21.3 |  | 119 | 38.9 |  | 2 | 0.8 |
| United States of America | 116 | 20.5 |  | 116 | 37.9 |  | 0 | 0.0 |
| Zimbabwe | 2 | 0.4 |  | 2 | 0.7 |  | 0 | 0.0 |
| More than one nationality | 3 | 0.5 |  | 3 | 1.0 |  | 0 | 0.0 |
| Not a nationality | 11 | 1.9 |  | 10 | 3.3 |  | 1 | 0.4 |
| NA | 20 | 3.5 |  | 11 | 3.6 |  | 6 | 2.3 |
| **Current or former student** |  |  |  |  |  |  |  |  |
| Yes | 458 | 80.8 |  | 222 | 72.5 |  | 236 | 91.5 |
| No | 106 | 18.7 |  | 84 | 27.5 |  | 22 | 8.5 |
| NA | 3 | 0.5 |  |  |  |  |  |  |
| **Highest level of education** |  |  |  |  |  |  |  |  |
| Compulsory school without vocational training | 15 | 2.6 |  | 10 | 3.3 |  | 5 | 1.9 |
| Compulsory school with vocational training | 13 | 2.3 |  | 9 | 2.9 |  | 4 | 1.6 |
| Vocational school with A-levels/high-school diploma | 59 | 10.4 |  | 41 | 13.4 |  | 18 | 7.0 |
| Academic High School/Grammar School | 180 | 31.7 |  | 40 | 13.1 |  | 140 | 54.3 |
| University/College | 295 | 52.0 |  | 206 | 67.3 |  | 89 | 34.5 |
| NA | 5 | 0.9 |  |  |  |  | 2 | 0.8 |
| **Currently or formerly employed** |  |  |  |  |  |  |  |  |
| Yes | 405 | 71.4 |  | 263 | 85.9 |  | 142 | 55.0 |
| No | 159 | 28.0 |  | 43 | 14.1 |  | 116 | 45.0 |
| NA | 3 | 0.5 |  |  |  |  |  |  |
|  |  |  |  |  |  |  |  |  |

### Figure S1

*Frequency Distribution of Raters Across Music Excerpts*

*
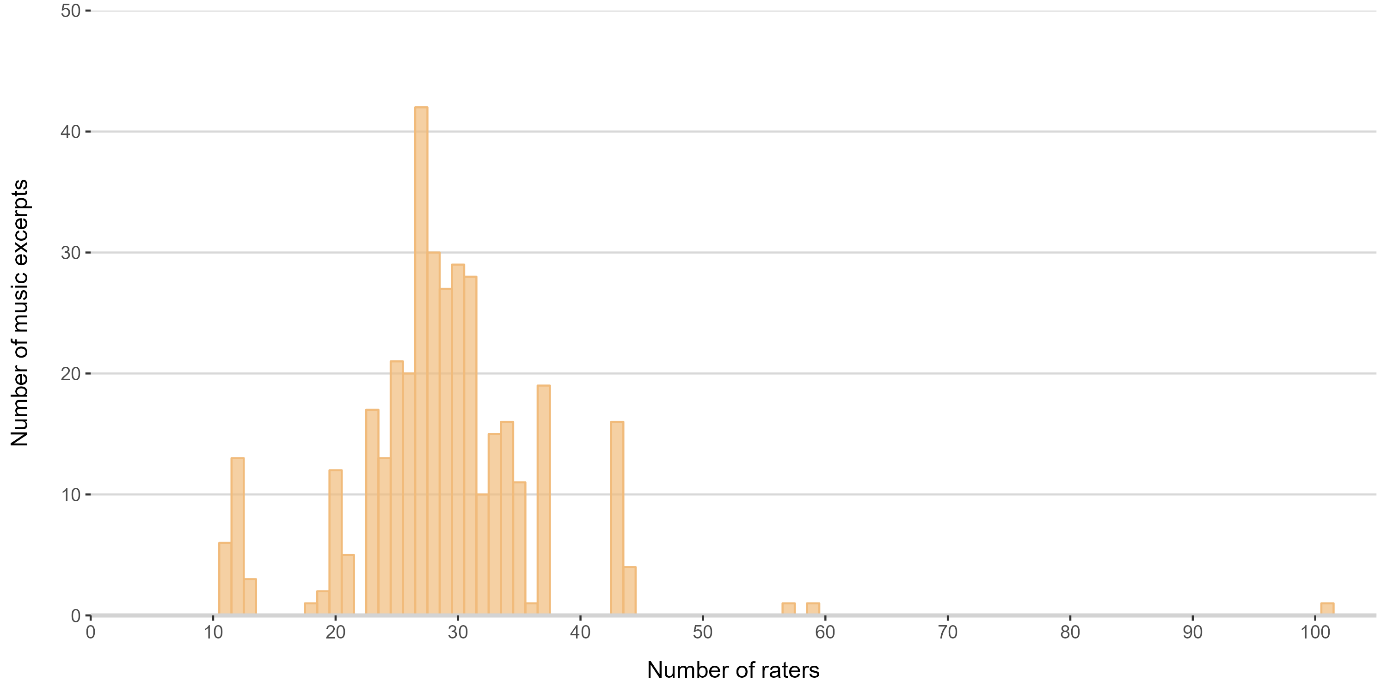
*

*Note.* A total of three songs had more than 50 raters as they were accidentally delivered multiple times instead of once.

### Table S2

*Top and Bottom 5% - Songs With Highest and Lowest Average Inter-Item Correlations (Dimension Level) across Genres*

| Code | Genre | *n* | *ICC* | Average interrater correlation | Title | | Artist | SUB | VIT | UNE | Familiarity  *M (SD)* | Liking  *M (SD)* |
| --- | --- | --- | --- | --- | --- | --- | --- | --- | --- | --- | --- | --- |
|  |  |  |  |  |  | |  |  |  |  |  |  |
| All songs |  | 28.76 | .80 | .20 |  | |  | 12.04 | 15.99 | 5.56 | 2.33 (1.15) | 3.13 (1.20) |
|  |  |  |  |  |  | |  |  |  |  |  |  |
| Top 5% |  | 29.06 | .94 | .43 |  | |  | 13.93 | 18.77 | 4.06 | 2.64 (1.00) | 3.36 (1.11) |
|  |  |  |  |  |  | |  |  |  |  |  |  |
| K096 | C | 23 | .93 | .48 | Preludes op. 32 no. 5 | | Sergei Rachmaninoff | 21.61 | 3.56 | 1.33 | 1.09 (0.42) | 3.26 (1.29) |
| H903 | H | 43 | .96 | .47 | Sad octopus | | Claptu feat. Clows | 18.67 | 1.74 | 1.84 | 1.23 (0.61) | 3.30 (1.19) |
| K085 | C | 26 | .96 | .47 | Symphony No. 8 (Finale) | | Anton Bruckner | 7.30 | 29.67 | 7.12 | 1.46 (0.65) | 3.42 (1.21) |
| P101 | P | 26 | .96 | .47 | Ex's & Oh's | | Elle King | 11.23 | 41.09 | 2.90 | 3.81 (1.47) | 3.88 (0.86) |
| K103 | C | 33 | .95 | .44 | Quartet, Op. 3 | | Alban Berg | 11.01 | 33.30 | 1.91 | 1.30 (0.85) | 1.85 (1.06) |
| K107 | C | 31 | .95 | .44 | O Fortuna (Carmina Burana) | | Carl Orff | 5.62 | 5.57 | 17.95 | 3.65 (1.38) | 3.61 (1.20) |
| P053 | P | 44 | .97 | .44 | Happy | | Pharrell Williams | 11.31 | 37.07 | 3.94 | 4.50 (1.05) | 3.73 (1.17) |
| H902 | H | 29 | .96 | .43 | Aurora | | Brillion | 21.86 | 2.64 | 1.77 | 1.48 (0.95) | 3.41 (0.87) |
| H904 | H | 25 | .94 | .43 | Aper | | Claptu feat. Beatmund Noise | 25.94 | 4.84 | 2.25 | 1.04 (0.20) | 3.36 (1.32) |
| K019 | C | 28 | .95 | .43 | Bénédiction de Dieu Dans la Solitude | | Franz Liszt | 7.64 | 26.98 | 1.60 | 1.43 (1.00) | 3.61 (1.20) |
| P042 | P | 28 | .94 | .43 | Fluorescent Adolescent | | Arctic Monkeys | 20.26 | 2.08 | 3.03 | 3.07 (1.74) | 3.96 (1.23) |
| P050 | P | 31 | .95 | .43 | I Wish | | Stevie Wonder | 12.64 | 26.58 | 1.71 | 2.29 (1.42) | 3.39 (1.05) |
| P003 | P | 12 | .89 | .42 | Heartbeats | | Jose Gonzales | 9.38 | 36.00 | 2.81 | 2.58 (1.44) | 4.33 (0.78) |
| P054 | P | 44 | .97 | .42 | Wannabe | | Spice Girls | 27.49 | 2.90 | 3.11 | 4.41 (1.04) | 3.84 (0.99) |
| H905 | H | 23 | .92 | .41 | Panther | | DECAP | 10.29 | 39.94 | 3.29 | 1.00 (0.00) | 1.74 (1.10) |
| P085 | P | 20 | .90 | .41 | Lush Life | | Zara Larsson | 20.22 | 3.96 | 2.20 | 2.90 (1.65) | 2.75 (1.16) |
| P092 | P | 34 | .96 | .41 | I Want It That Way | | Backstreet Boys | 9.95 | 23.52 | 0.98 | 4.53 (0.93) | 3.65 (1.20) |
| P136 | P | 31 | .96 | .41 | Uptown Funk | | Mark Ronson | 4.19 | 11.81 | 15.70 | 4.29 (1.13) | 3.45 (1.41) |
| P086 | P | 21 | .92 | .40 | Everybody | | Backstreet Boys | 8.12 | 23.31 | 1.78 | 4.10 (1.04) | 3.29 (0.85) |
|  |  |  |  |  |  | |  |  |  |  |  |  |
| Bottom 5% | | 22.53 | 0.31 | .03 | |  |  | 11.92 | 11.40 | 7.09 | 2.19 (1.17) | 2.94 (1.25) |
|  | |  |  |  | |  |  |  |  |  |  |  |
| K005 | C | 12 | .34 | .05 | Piano Sonata BB 88 (Sz. 80) | | Béla Bartók | 11.79 | 14.41 | 6.73 | 2.08 (1.31) | 2.75 (1.22) |
| K112 | C | 29 | .64 | .05 | Brandenburg Concertos | | J. Sebastian Bach | 11.94 | 18.21 | 5.46 | 1.66 (1.23) | 2.76 (1.48) |
| K001 | C | 13 | .43 | .04 | A Midsummer Night's Dream | | Felix Mendelssohn | 10.91 | 14.56 | 0.94 | 2.08 (1.19) | 3.46 (0.88) |
| K007 | C | 11 | .42 | .04 | Prince Igor (Chorus) | | Alexander Borodin | 16.47 | 10.97 | 2.48 | 2.09 (1.38) | 3.00 (1.26) |
| K093 | C | 20 | .59 | .04 | The Four Seasons (Spring) | | Antonio Vivaldi | 11.57 | 17.71 | 4.13 | 3.40 (1.35) | 2.80 (1.32) |
| P047 | P | 27 | .59 | .04 | Redbone | | Childish Gambino | 12.08 | 10.91 | 5.78 | 2.67 (1.64) | 3.11 (1.34) |
| P083 | P | 23 | .51 | .04 | The Village | | Wrabel | 17.24 | 8.79 | 10.24 | 2.04 (1.40) | 3.43 (1.24) |
| K045 | C | 27 | .19 | .03 | Piano Sonata No. 3 | | Johannes Brahms | 11.00 | 16.19 | 9.29 | 1.96 (1.40) | 3.22 (1.12) |
| K046 | C | 31 | .40 | .03 | Messiah (Hallelujah) | | Georg F. Händel | 9.92 | 13.43 | 6.87 | 1.94 (1.29) | 2.81 (1.40) |
| P008 | P | 35 | .57 | .03 | Your Song | | Elton John | 16.73 | 14.12 | 9.32 | 4.29 (1.02) | 4.09 (0.92) |
| P119 | P | 25 | .36 | .03 | I Am I Said | | Neil Diamond | 7.29 | 7.98 | 2.46 | 1.72 (1.17) | 2.52 (0.96) |
| P088 | P | 21 | .44 | .02 | Hide and Seek | | Imogen Heap | 9.81 | 3.46 | 11.26 | 2.05 (1.60) | 2.71 (1.35) |
| P100 | P | 33 | .45 | .02 | Halo | | Beyonce | 14.46 | 18.60 | 8.12 | 4.09 (1.01) | 3.36 (1.32) |
| H008 | H | 34 | .17 | .01 | Laura | | Prinz Pi | 6.31 | 5.80 | 11.55 | 1.24 (0.70) | 2.18 (1.11) |
| H048 | H | 25 | .07 | .01 | Leaving the past | | Immortal Technique | 8.92 | 9.19 | 11.98 | 1.00 (0.00) | 2.64 (1.41) |
| H077 | H | 32 | -.13 | .01 | Too Much | | Drake | 11.50 | 11.62 | 6.34 | 1.50 (0.84) | 2.84 (1.25) |
| K008 | C | 12 | .01 | .01 | Gremin's Aria (Onegin) | | Pyotr I. Tchaikovsky | 13.83 | 7.41 | 4.98 | 2.33 (1.67) | 2.92 (1.62) |
| K082 | C | 26 | .13 | .01 | Var det en dröm? | | Jean Sibelius | 13.21 | 6.16 | 12.39 | 1.23 (0.71) | 2.58 (1.21) |
| K009 | C | 11 | -.25 | .00 | Kol Nidrei, Op. 47 | | Max Bruch | 11.51 | 7.07 | 4.34 | 2.18 (1.33) | 2.73 (1.35) |

*Note. n* = number of raters per excerpt; SUB = Sublimity; VIT = Vitality; UNE = Unease; C = Classical; H = Hip-hop/Rap; P = Pop.

**Figure S2**

Mean Frequencies of Percent of Participants Who Selected Different Emotion Terms for a Given Musical Excerpt (Bars) and Mean Intensities of the Different Emotion Terms Across Musical Excerpts (line)


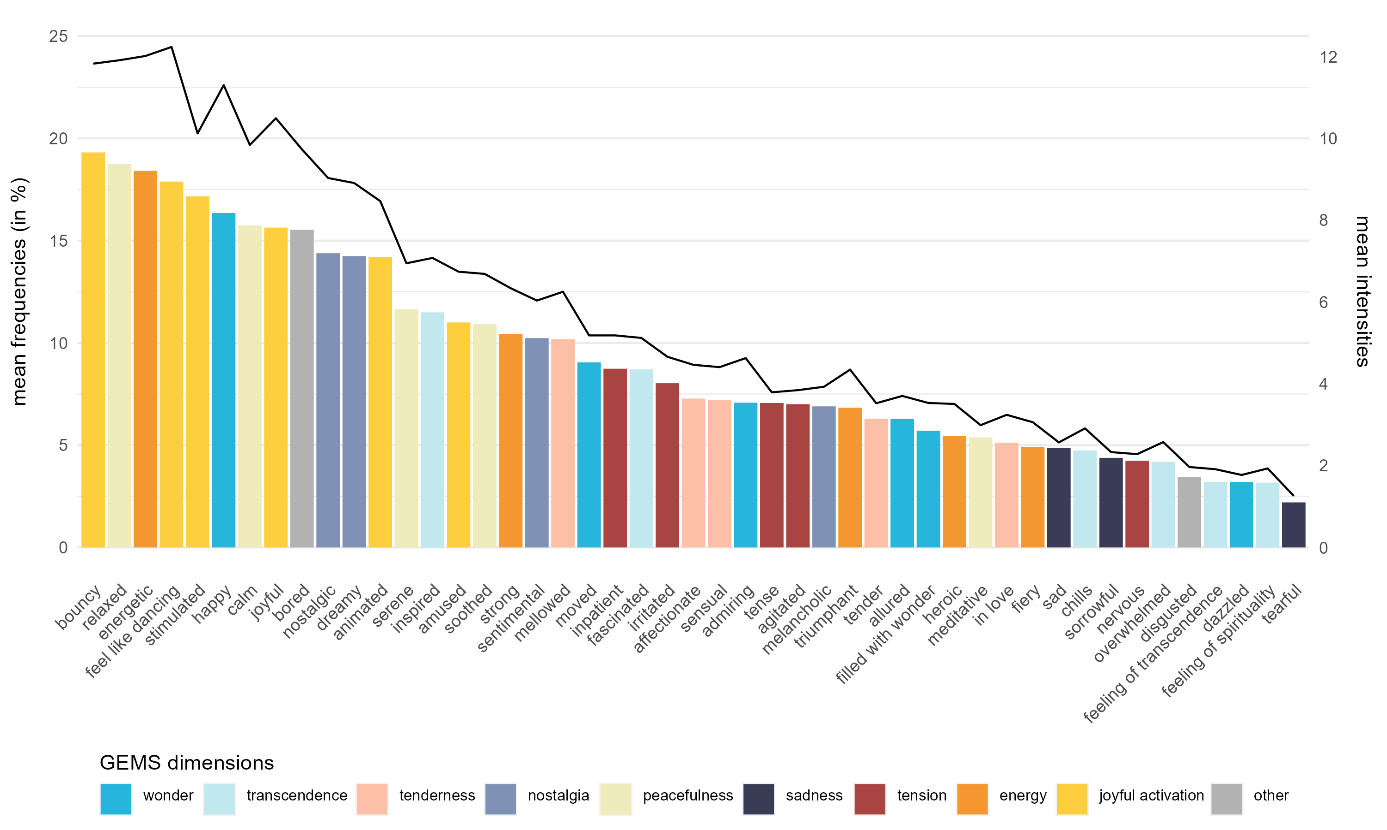


### Table S3

Descriptive Summary of the Intensities of the GEMS Dimensions and Their Factor Means

|  | M | SD | Mdn | Min | Max |
| --- | --- | --- | --- | --- | --- |
| Sublimity | 12.04 | 5.91 | 11.32 | 2.09 | 29.51 |
| Wonder | 13.22 | 6.06 | 13.14 | 0.00 | 37.21 |
| Transcendence | 9.44 | 5.33 | 8.55 | 0.00 | 26.42 |
| Tenderness | 9.42 | 7.69 | 7.56 | 0.00 | 34.97 |
| Nostalgia | 13.10 | 9.13 | 12.40 | 0.00 | 40.71 |
| Peacefulness | 15.01 | 10.18 | 13.20 | 0.00 | 48.16 |
|  |  |  |  |  |  |
| Vitality | 15.99 | 8.86 | 15.43 | 0.78 | 41.09 |
| Energy | 12.21 | 8.92 | 10.51 | 0.00 | 47.34 |
| Joyful activation | 19.76 | 10.58 | 18.61 | 0.00 | 51.04 |
|  |  |  |  |  |  |
| Unease | 5.67 | 3.68 | 4.78 | 0.00 | 20.04 |
| Sadness | 3.21 | 4.88 | 1.39 | 0.00 | 29.94 |
| Tension | 8.13 | 6.73 | 6.22 | 0.00 | 34.79 |

*Note*. Values are based on all 364 songs.

### Table S4

*Inter-Correlations and Internal Consistencies for GEMS Dimension and Factor Mean Intensities Across All Music Excerpts*

|  | 1 | 2 | 3 | 4 | 5 | 6 | 7 | 8 | 9 | 10 | 11 | 12 |
| --- | --- | --- | --- | --- | --- | --- | --- | --- | --- | --- | --- | --- |
| (1) Wonder | (.54) |  |  |  |  |  |  |  |  |  |  |  |
| (2) Transcendence | .42** | (.57) |  |  |  |  |  |  |  |  |  |  |
| (3) Tenderness | .37** | .30** | (.60) |  |  |  |  |  |  |  |  |  |
| (4) Nostalgia | .47** | .27** | .69** | (.54) |  |  |  |  |  |  |  |  |
| (5) Peacefulness | .26** | .28** | .78** | .62** | (.65) |  |  |  |  |  |  |  |
| (6) Energy | .12* | .08 | -.51** | -.36** | -.56** | (.67) |  |  |  |  |  |  |
| (7) Joyful activation | .27** | -.21** | -.39** | -.20** | -.41** | .65** | (.72) |  |  |  |  |  |
| (8) Sadness | .16** | .26** | .40** | .54** | .30** | -.36** | -.46** | (.63) |  |  |  |  |
| (9) Tension | -.38** | -.02 | -.55** | -.58** | -.54** | .24** | -.06 | -.23** | (.64) |  |  |  |
| (10) Sublimity | .61** | .53** | .87** | .85** | .84** | -.40** | -.29** | .46** | -.59** | (.79) |  |  |
| (11) Vitality | .22** | -.09 | -.49** | -.30** | -.53** | .89** | .92** | -.45** | .08 | -.37** | (.77) |  |
| (12) Unease | -.24** | .15** | -.24** | -.17** | -.29** | -.02 | -.36** | .46** | .76** | -.24** | -.22** | (.64) |

*Note*. *N* = 364; Values in parentheses indicate McDonald’s *ω*.

* *p* < .05. ** *p* < .01.

## Genre Differences in Mean Intensities of Emotions Experienced

The strongest differences were found for *Transcendence* and *Nostalgia*, with *F*(2,361) = 92.66 and *F*(2,361) = 49.71 (all *ps* < .001) respectively. Mean intensities of *Transcendence* were significantly higher in classical music (*M* = 14.29, *SD* = 4.48) compared to hip-hop/rap (*M* = 7.13, *SD* = 4.18) and pop (*M* = 7.76, *SD* = 4.39). For *Nostalgia*, all three genres were found to have significant mean differences, with the highest intensities in pop (*M* = 17.38, *SD* = 8.85), followed by classical music (*M* = 13.97, *SD* = 8.69) and hip-hop/rap (*M* = 7.39, *SD* = 6.54). It is also notable that feelings of tension were significantly less pronounced in pop (*M* = 4.69, *SD*= 3.22), compared to hip-hop/rap (*M* = 11.30, *SD* = 6.51) and classical music (*M* = 9.06, *SD*= 8.21). Out of all nine dimensions, *Tension* actually proved to be most strongly associated with the average liking of the respective songs (*r* = -.61, *p* < .001). Yet, significant mean differences in *Tension* between pop and the other two genres even held up when controlling for average liking in a one-way ANCOVA, with *F*(2, 360) = 9.68 (*p* < .001).

### Table S5

*Means, Standard Deviations and ANOVA results with Tukey Post-Hoc-Test for Overall Dimension and Factor Intensities Across All Music Excerpts by Genre*

|  | Classical music | |  | Hip-hop/Rap | |  | Pop | |  | Mean Differences | | |  |  | |
| --- | --- | --- | --- | --- | --- | --- | --- | --- | --- | --- | --- | --- | --- | --- | --- |
|  | *M* | *SD* |  | *M* | *SD* |  | *M* | *SD* |  | C-H | P-H | P-C |  | *F*(2,361) | |
| **Sublimity** | **14.60** | **5.90** |  | **7.95** | **4.67** |  | **13.63** | **4.97** |  | **6.65**** | **5.69**** | **-0.97** |  | **57.25**** | |
| Wonder | 14.97 | 4.27 |  | 9.29 | 5.09 |  | 15.30 | 6.35 |  | 5.68** | 6.01** | 0.34 |  | 47.52** | |
| Transcendence | 14.29 | 4.48 |  | 7.13 | 4.18 |  | 7.76 | 4.39 |  | 7.16** | 0.63 | -6.53** |  | 92.66** | |
| Tenderness | 11.05 | 8.03 |  | 5.11 | 5.65 |  | 11.91 | 7.40 |  | 5.94** | 6.81** | 0.86^+^ |  | 33.72** | |
| Nostalgia | 13.97 | 8.69 |  | 7.39 | 6.54 |  | 17.38 | 8.85 |  | 6.57** | 9.99** | 3.42** |  | 49.71** | |
| Peacefulness | 18.74 | 12.38 |  | 10.82 | 9.51 |  | 15.81 | 7.25 |  | 7.92** | 4.99** | -2.93^+^ |  | 19.44** | |
|  |  |  |  |  |  |  |  |  |  |  |  |  |  | |  |
| **Vitality** | **13.84** | **8.97** |  | **16.52** | **7.89** |  | **17.15** | **9.33** |  | **-2.69^+^** | **0.63** | **3.32*** |  | **4.60*** | |
| Joyful activation | 12.32 | 10.48 |  | 13.25 | 8.44 |  | 11.23 | 7.94 |  | -0.94 | -2.02 | -1.08 |  | 1.67 | |
| Energy | 15.36 | 9.09 |  | 19.79 | 8.85 |  | 23.07 | 11.79 |  | -4.43** | 3.28* | 7.71** |  | 17.31** | |
|  |  |  |  |  |  |  |  |  |  |  |  |  |  |  | |
| **Unease** | **6.35** | **4.30** |  | **6.53** | **3.51** |  | **4.42** | **2.92** |  | **-0.18** | **-2.11**** | **-1.93**** |  | **14.05**** | |
| Sadness | 3.64 | 4.62 |  | 1.76 | 3.29 |  | 4.14 | 5.87 |  | 1.88* | 2.39** | 0.50 |  | 8.59** | |
| Tension | 9.06 | 8.21 |  | 11.30 | 6.51 |  | 4.69 | 3.22 |  | -2.25* | -6.61** | -4.37** |  | 39.39** | |

*Note. N* = 364.

^+^ p < .10. * p < .05. ** *p* < .01.

## Order of Songs and Emotions Experienced

In summary, we detected only very weak correlations, of which few were significant. For example, in descending order of effect size, negative correlations were found between performance order and the GEMS items bouncy (*r =*-.04, p < .001), mellowed (*r* = -.03, *p* = .002), relaxed (*r* = -.03, *p* = .002), joyful (*r* = -.03, *p* = .004), feel like dancing (*r* = -.03, *p* = .005) and soothed (*r* = -.03, *p* = .006), indicating that these items were selected less often when a piece was presented later. At the dimension and factor level, we found negative correlations between performance order and Vitality (*r* = -.03, *p* < .001) and Peacefulness (*r* = -.02, *p* = .013). The only significant positive correlation appeared with the items bored (*r* = .04, *p* < .001) and melancholic (*r* = .02, *p* = .018). These items were thus slightly more likely to be felt when a piece was presented later. However, since all correlations were very weak, the order does not seem to matter too much, at least when 10-12 pieces of music are presented.
